# Indigenous identity identification in administrative health care data globally: A scoping review

**DOI:** 10.1177/13558196231219955

**Published:** 2023-12-15

**Authors:** Mandi Gray, Kienan Williams, Richard T. Oster, Grant Bruno, Annelies Cooper, Chyloe Healy, Rebecca Rich, Shayla Scott Claringbold, Gary Teare, Samara Wessel, Rita I. Henderson

**Affiliations:** 1 Department of Family Medicine, Cumming School of Medicine, 70401 University of Calgary, Calgary, AB, Canada; 2 Indigenous Wellness Core, 3146 Alberta Health Services, Canada, Edmonton, AB, Canada; 3 Department of Pediatrics, 3158 University of Alberta, Edmonton, AB, Canada; 4 Indigenous Health and Environmental Justice, Critical Health and Social Action Lab., 113749 University of Toronto, Toronto, ON, Canada; 5 661630 Blackfoot Confederacy Tribal Council, Calgary, AB, Canada; 6 3158 University of Alberta, Edmonton, AB, Canada; 7 Community Health Sciences, Cumming School of Medicine, University of Calgary, Calgary, AB, Canada; 8 Provincial Population and Public Health, 3146 Alberta Health Services, Calgary, AB, Canada; 9 Counselling Psychology, Department of Educational Psychology, 2129 University of Calgary, Calgary, AB, Canada

**Keywords:** health data, Indigenous identification, Indigenous health

## Abstract

**Objective:**

Both Indigenous and non-Indigenous governments and organizations have increasingly called for improved Indigenous health data in order to improve health equity among Indigenous peoples. This scoping review identifies best practices, potential consequences and barriers for advancing Indigenous health data and Indigenous data sovereignty globally.

**Methods:**

A scoping review was conducted to capture the breadth and nature of the academic and grey literature. We searched academic databases for academic records published between 2000 and 2021. We used Google to conduct a review of the grey literature. We applied Harfield’s Aboriginal and Torres Strait Islander Quality Appraisal Tool (QAT) to all original research articles included in the review to assess the quality of health information from an Indigenous perspective.

**Results:**

In total, 77 academic articles and 49 grey literature records were included. Much of the academic literature was published in the last 12 years, demonstrating a more recent interest in Indigenous health data. Overall, we identified two ways for Indigenous health data to be retrieved. The first approach is health care organizations asking clients to voluntarily self-identify as Indigenous. The other approach is through data linkage. Both approaches to improving Indigenous health data require awareness of the intergenerational consequences of settler colonialism along with a general mistrust in health care systems among Indigenous peoples. This context also presents special considerations for health care systems that wish to engage with Indigenous communities around the intention, purpose, and uses of the identification of Indigenous status in administrative databases and in health care settings. Partnerships with local Indigenous nations should be developed prior to the systematic collection of Indigenous identifiers in health administrative data. The QAT revealed that many research articles do not include adequate information to describe how Indigenous communities and stakeholders have been involved in this research.

**Conclusion:**

There is consensus within the academic literature that improving Indigenous health should be of high priority for health care systems globally. To address data disparities, governments and health organizations are encouraged to work in collaboration with local Indigenous nations and stakeholders at every step from conceptualization, data collection, analysis, to ownership. This finding highlights the need for future research to provide transparent explanation of how meaningful Indigenous collaboration is achieved in their research.

## Introduction

Within the last decade, Indigenous communities, leaders, and researchers have expressed a need for improved access to valid and accurate Indigenous health data specific to their nations and communities.^1,2^ Globally, there is a general lack of Indigenous specific disaggregated data, with one of the major barriers being that many countries have difficulty identifying the Indigenous population in administrative health data.^2,3^ Despite countries such as Canada, Australia, New Zealand and US being global leaders when it comes to data on Indigenous populations, there are still numerous challenges including inadequate Indigenous representation in national surveys, identification of Indigenous peoples in administrative datasets, defining Indigenous identity, and concerns regarding data governance practices.^3^ In comparison to non-Indigenous people, there is far less Indigenous specific administrative health.^1,4^ Indigenous and non-Indigenous governments and organizations alike have called for improved Indigenous health data in order to improve health equity among Indigenous peoples.^5,6^

Data are a powerful tool that can be used to benchmark health care status, demonstrate need, and also to measure progress.^3^ The absence of accessible, valid, and reliable Indigenous health data has numerous consequences for Indigenous peoples health – such as the underestimation of inequities in health determinants, population health status and health care access.^7^ Improved Indigenous health data could also be used to demonstrate progress in closing health gaps – for example, by demonstrating improvements in Indigenous health in vaccine coverage and cardiovascular deaths.^8^

Indigenous involvement and governance over their health data is also a paramount concern. Historically, Indigenous nations and peoples have been excluded from providing input into large-scale surveys and research.^3^ Beyond exclusion, researchers and government organizations have historically used Indigenous peoples as test subjects to advance medical research without their informed consent.^9–11^ The historical context has resulted in widespread distrust amongst Indigenous peoples globally of researchers, universities, and governments. Furthermore, the health data that has been collected is most often dictated by the priorities of non-Indigenous governments and health care organizations that may not reflect the specific health priorities of Indigenous nations. Another problematic element is that colonizers have historically had the power to categorize and classify Indigenous identity, which may not necessarily be relevant to the Indigenous nations.^4^ Finally, Indigenous nations have raised concerns about enhanced data due to previous research and government initiatives that have caused considerable harm for their people. There are also concerns based on past incidents that the dissemination of Indigenous health data could be weaponized by non-Indigenous people and institutions to pathologize and stigmatize Indigenous peoples.^12^

In light of these issues the objective of this scoping review was to describe best practices, potential consequences and barriers for aligning Indigenous health data collection with Indigenous community priorities. This review includes two potential possibilities for Indigenous identification through data linkage and voluntary self-identification in health care settings.

The scoping review was guided by two questions:

1. What is known about data collection about Indigenous identities in health care settings?
2. What is known about Indigenous health care user data globally?

These overlapping questions were deliberately broad to ensure that relevant studies were not excluded prior to gaining an understanding of the literature.^13^

## Methods

The scoping review core research team included First Nations (KW) and non-Indigenous (MG, RH, RO) academics and health care service researchers located within university and health services settings. The research was guided by a Research Advisory Committee comprised of six members, four of whom are Indigenous and two who are non-Indigenous. All the Research Advisory Committee members were invited to join because of their unique expertise in Indigenous health care. The intention of the advisory committee was to ensure accountability in the research process and that the research would be beneficial to local Indigenous nations, tribal councils and decision makers, as well as providing guidance on the methodology and analysis. The core research team and the advisory committee met on two occasions to discuss the scoping review methodology and to coanalyse the findings of the review. All advisory members were invited to be coauthors on the paper.

Arksey and O’Malley’s (2005) scoping review methodology was used.^13^ This comprises six steps: (1) research question identification, (2) identification of relevant studies, (3) study selection, (4) charting data, (5) collecting, summarizing and reporting results, and (6) consultation with stakeholders. In steps four and five, we incorporated a thematic analysis to organize and understand the data.^14^

### Search strategy

The databases searched included: Scholar, MEDLINE/PubMed, CINAHL, Jstor, Proquest and iPortal. These databases were identified in collaboration with an Alberta Health Services librarian. The databases were selected because they included relevant academic literature across disciplines including medicine, health services research, Indigenous studies and social sciences. The searches of the academic literature were conducted in November 2021. We also reviewed reference lists of included sources for additional sources that met the inclusion criteria.

We kept the inclusion criteria intentionally broad, namely, articles of any geographic region published in peer reviewed journals utilizing any methods, limiting reviews included to those written in English. All the literature was published between a preestablished period of 2000–2021, to ensure relative currency. The first author (MG) in collaboration with an Alberta Health Services librarian conducted a preliminary review of records and excluded any that clearly did not meet the study criteria (i.e. minimal focus on health care user data and Indigenous identity). Two authors (MG and AC) then reviewed all the remaining study abstracts to build consensus around if a source assisted with answering the research questions. This began with MG and AC independently reviewing all abstracts, which then involved a full text review if either team member were uncertain about whether a given source was relevant to the research questions. The reference lists of all the included articles were scanned for additional relevant sources. There was a second round of inclusion and exclusion assessment based on the publication abstract and charting the data from these references. MG and AC then came to consensus on which sources to include.

An accompanying grey literature search was conducted in June 2022, focussing on Indigenous health data from the past 5 years. The search was confined to results from the first 10 result pages of a Google search. A total of 130 articles were identified, out of which 49 were included in the review. The grey literature was reviewed independently by one author (SW) to determine relevance, articles deemed relevant or possibly relevant were then reviewed by the first author (MG) and included in the review. Due to space constraints, we do not focus on the grey literature in the remainder of this article. Nevertheless, the grey literature was useful in helping us consider the academic literature more deeply.

### Data charting, summarization and reporting

We developed a data extraction chart in Microsoft Excel. Key data extracted included publication year, country, methods, key findings, data governance and main argument. Data were compiled for synthesis by two authors (MG, AC) with the help of a research assistant. The two authors met throughout the extraction process to review data and discuss findings. As themes were identified and developed understanding of the literature, we excluded studies that did not adequately assist with answering the overarching research questions. Examples of excluded articles were:

• Those addressing race and sociodemographic data in health care settings generally, without specifying considerations for Indigenous populations or explicitly discussing Indigenous health data collection.
• Those that engaged in collecting Indigenous identity data or used data matching but the research did not engage in substantive discussion about the identification of Indigenous peoples in datasets beyond the methods section.
• Those that reported on the prevalence of certain conditions among Indigenous peoples but did not provide substantive description of the methods used to identify Indigenous populations.

Ultimately, we included 77 academic articles and 49 grey literature articles (our search process is described below). The list of included academic articles is given as S1 in the online supplement. Those included articles we discuss in this article are also given in the reference list at the end of this article.

Once all the data had been charted, we organized the academic sources into four themes – (1) Policy recommendations, (2) Indigenous health surveys, census, birth and mortality data collection, (3) Data linkage and algorithm development, and (4) Indigenous and racial identity data collection in health care settings. Most of the articles provided policy level recommendations for health care systems but only articles that exclusively examined policy were coded as ‘Policy recommendations’. The themes were identified collectively by authors (MG, AC) after closely reviewing the included academic literature. The academic articles, categorized by theme, are given as S2 in the online supplement.

The list of the included grey literature articles is given as S3 in the online supplement.

### Cultural quality appraisal tool

At the request of the Research Advisory Committee, we conducted a cultural appraisal of the included academic articles using Harfield’s Aboriginal and Torres Strait Islander Quality Appraisal Tool (QAT).^15^ Regarding research into Aboriginal and Torres Strait Islanders, the QAT appraises research quality from an Indigenous perspective to assess how Aboriginal and Torres Strait Islander peoples lead, govern and are included in the research process. The QAT was developed to respond to the lack of attention to Indigenous epistemologies, values and principles in primary health research.^15^ We applied the QAT to the academic research in this study. Two authors (MG and SW) with the help of a research assistant reviewed all the research articles (*n* = 46) included in the scoping review, excluding commentaries. The two authors reviewed each article independently to assess each of the 14 questions of the QAT to provide an answer of Yes, Partial or No, which were then scored (Yes = 1, Partial = 0.5, No = 0). The two authors then came to a consensus regarding the scoring of each article.

### Critical dialogues

The final step was to critically discuss the scoping review findings with the core research team and Research Advisory Committee. The process was influenced by Chambers’ (2018) suggestion for ‘dialoguing with the tensions’, which seek to unpack the epistemological tensions between Western and decolonizing knowing between decolonizing knowing and Western ways of doing scoping reviews.^16^ Dialoguing with the tensions is a process of reflexivity engaged with by the research team to identify those tensions between Western ways of knowing and Indigenous ways of knowing. To engage in a reflexive dialogue about the findings, MG presented the findings to the Research Advisory Committee, a First Nation health board, senior management in the Indigenous Wellness Core within Alberta Health Services and the Wisdom Council (a group of diverse Indigenous people, Knowledge Keepers and Elders from across Alberta who provide guidance to Alberta Health Services). At the conclusion of each presentation, the attendees were asked for their interpretation of the findings or any key considerations to be addressed by the research. These discussions centred around the tensions between academic literature and health care services with the principles of Indigenous data sovereignty that guided our overarching objectives in pursuing this research. Feedback from these presentations has nurtured dialogue about the systemic collection of Indigenous status in health care settings and supported the analysis of the findings.

## Results

**Figure 1.**
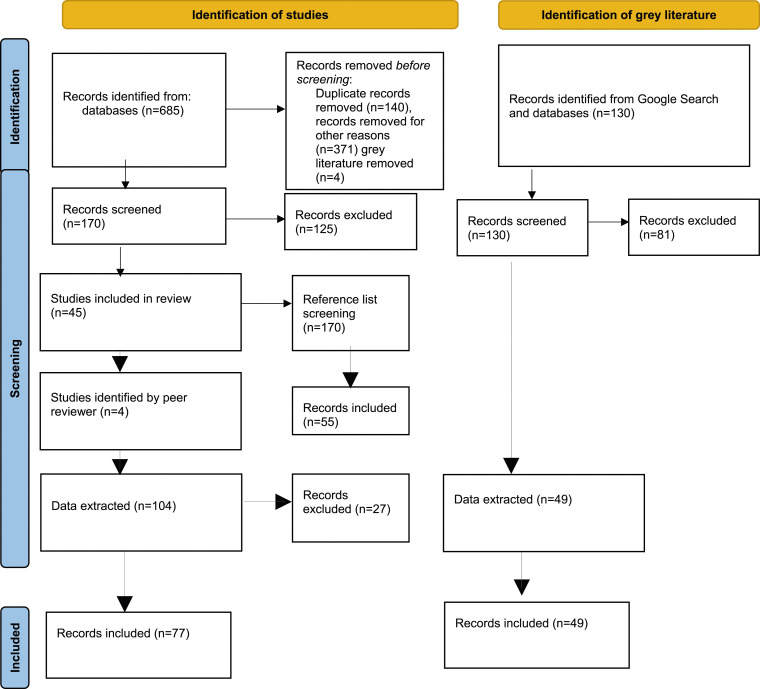
PRISMA flow diagram.

As shown in Figure 1, 685 academic records were retrieved from the initial peer-reviewed database search. Prior to screening for inclusion, we removed duplicate records (*n* = 140) and irrelevant records (*n* = 375), leaving 170 records to be screened. Of these, 125 were excluded, leaving 45 peer-review articles included. The reference list screening yielded 55 additional records. Four articles were further identified by a peer reviewer, resulting in a total inclusion of 104 records. We collectively decided to exclude 27 articles that did not adequately address the research questions because they did not substantively examine Indigenous identification in health care settings. These articles were initially included as they may have referenced the issues of Indigenous identification but upon review did not substantively examine the issue specific to the Indigenous population.

Ultimately, the scoping review included 77 academic articles and 49 pieces of grey literature.

### General characteristics

The general characteristics of the academic articles are reported in Table 1. Much of the literature (75.4%) was published between 2011 and 2021. Nearly half (46.8%) of the articles relied on quantitative methods, often to assess the reliability and validity of various Indigenous health identifiers in administrative databases including data linkage and algorithm development. Almost half (41.6%) of the articles were based in Australia, 23.4% in the US, 15.6% in Canada, 11.7% were international in scope (most frequently focussing on data efforts in the previously mentioned countries given the similarity of advanced statistical systems and a shared history of colonialism), a small number of articles (3.9%) were from Aotearoa – New Zealand. There was only one article each for Bolivia, Mexico and Norway.

**Table 1.** General characteristics of included academic articles.

Characteristics	Number (*n* = 77)	Percentage (%)
Publication year		
2017 – 2021	32	41.6
2011 – 2016	26	33.8
2005 – 2010	16	20.8
<2004	3	3.9
Study design		
Quantitative methods	36	46.8
Commentary	24	31.2
Qualitative methods	10	13.0
Mixed methods	3	3.9
Evaluation	2	2.6
Historical analysis	1	1.3
Policy review	1	1.3
Country		
Australia	32	41.6
United States	18	23.4
Canada	12	15.6
International^a^	9	11.7
Aotearoa – New Zealand	3	3.9
Bolivia	1	1.3
Mexico	1	1.3
Norway	1	1.3

^a^Defined as including more than two countries.

A summary of the characteristics of the grey literature is given as Table 2. Again, much of the literature (69.4%) was published in 2011–2021. The single largest topic area was Indigenous identifier. Most of the articles were based in Canada or Australia.

**Table 2.** General characteristics of included grey literature.

Characteristics	Number (*n* = 49)	Percentage (%)
Publication year		
2017 – 2022	21	42.9
2011 – 2016	13	26.5
2005 – 2010	10	20.4
<2004	2	4.1
No date	3	6.1
Study topic		
Indigenous identifier	11	22.4
Indigenous health	9	18.4
Data collection	6	12.2
Health data	6	12.2
Data governance	4	8.2
Data linkage	3	6.1
Data principles/protocols	3	6.1
Health indicators	2	4.1
Indigenous/ethnicity data	2	4.1
Mortality database	1	2.0
Data sharing	1	2.0
Disaggregated data	1	2.0
Country		
Canada	22	44.9
Australia	18	36.7
Aotearoa – New Zealand	6	12.2
International^a^	2	4.1
United States	1	2.0

^a^Defined as including more than two countries.

The literature represents a wide array of approaches to measure, collect and analyze Indigenous health data. Overall, two primary methods for Indigenous identification were addressed within the academic and grey literature: voluntary self-identification of Indigenous identity and data linkage. We discussed this in detail below.

Both approaches have strengths and limitations. Beyond methods for identification is critical discussions about how Indigenous identity is defined and conceptualized, which differs between non-Indigenous governmental organizations and how Indigenous nations may define membership within their nations. There are also specific historical, legal and cultural contexts that result in various challenges to identifying Indigenous peoples in health care data. For example, Canada does not have a consistent Indigenous identifier in their databases and many large scale health surveys do not have sufficient Indigenous representation in the sampling methodology to produce reliable disaggregated estimates.^7^ In contrast, Australia implemented the Standard Indigenous Question in 1996, which requires every person to be asked if they identify as Aboriginal or Torres Strait Islander in censuses or surveys by health, social or educational service providers, in registries and when accessing services.^17^ In Nordic countries, as a means to combat risk for discrimination in public services, legislation forbids the collection of race or Indigenous identity data.^18^ However, some have argued that by not collecting such data, discrimination may nevertheless persist due to lack of population-specific data that could more precisely identify health care inequities and better guide service provision.^19^

### Defining Indigenous identity

Indigenous identity is complex, fluid and more nuanced than simply including a checkbox based on blood quantum or genealogy alone.^19,20^ Indigenous identity is a deeply personal experience that is shaped by colonialism as well as social, emotional, political and financial forces.^19^ Indigenous scholars and communities have argued that Indigenous identification should also include kinship and community relations.^4^ The United Nations Permanent Forum on Indigenous Issues states that self-identification requires that the individual is accepted by the community as a member.^4^ This approach can be challenging due to colonial policies and practices, such as residential schools and forced adoption of Indigenous children, which have removed Indigenous people from their traditional territories and have severed kinship ties.^4^

Due to colonialism, Indigenous peoples have long been classified and labelled by settler colonial society and structures. In the spirit of reconciliation, Indigenous nations need the authority to define nation membership.^4^ For example, Andersen (2016) argues that data collection about Métis people in Canada often relies on a colonial logic that fundamentally misunderstands Métis identity and that for meaningful data collection to occur, terms and approaches should be more attentive to the complexity of Métis history.^21^ One suggestion Andersen makes is to ask those who chose to self-identify as Métis to also answer a sub-question specifically about Métis organizational attachment, to be able to identify those who have been formally recognised by the Métis community.^22^

### Voluntary Indigenous identity disclosure in health care settings

This is the first of the two primary methods for Indigenous identification discussed in the literature. Twenty-two articles examined Indigenous identity collection in health care settings. Many of the articles identified challenges of reliable collection of an Indigenous identifier in health care settings. One major barrier stems from Indigenous peoples’ distrust of the health care system, which may make them hesitant to disclose Indigenous identity out of concern that they will be discriminated against.^22–24^

Indigenous people were frequently misclassified or under-identified by health care staff, resulting in data that was often incomplete and inaccurate.^25,26^ The misclassification and under-identification was often tied to a lack of training among health care staff about the importance of routine data collection.^27–29^ Furthermore, in many cases, there is no identifier at all or when there is one, it is inconsistent across datasets.^3,8^

Emergency Departments (ED.)/Ambulatory Care and Primary Care were the most examined health care settings in the literature. The research demonstrates numerous barriers to collecting accurate and relevant Indigenous identity data in these settings, often tied to software constraints that restrict introducing a new data point, inconsistency in health care reporting, and health care service provider perceptions and attitudes towards relevance of collecting an Indigenous identifier. Interviews with key informants at a site that had implemented an Indigenous identifier noted several factors that influenced the success of the implementation, including supportive hospital executive staff and a high level of engagement with local Indigenous communities in project design and implementation.^22^

An evaluation of Indigenous identity collection in Australian Eds found varying degrees of accuracy of Indigenous identity reporting.^22^ Accuracy was calculated by the recording of Indigenous identity in the ED. information system divided by the ‘expected’ number of ED. visits by Indigenous peoples as determined by the Enhanced Reporting of Aboriginality variable in the statutory public health and disease register. The accuracy among the participating Eds ranged from 45.5% to 87.2% with an average of 76%. Another study of an Australian ED. found that it was more common for Indigenous peoples to be under-identified than over-identified.^30^ An Australian study of Indigenous identity collection in primary care settings found that nearly 20% of Indigenous patients did not have their status recorded in their clinical record suggesting that there was a systemic problem of failing to collect Indigenous identity data.^31^ Health care systems are encouraged to provide staff training on the multilayered benefits of systematically tracking Indigenous identification among clients.^32,33^

Despite Australia having legislation requiring the collection of Aboriginal and Torres Strait Islander identification, Indigenous status remains frequently underreported.^22,30^ Research has found that many health care practitioners in Australia do not systematically collect Indigenous identity information.^28,33^ A study of Indigenous identity collection in primary care settings in Australia found that higher rates of identifier recording were often associated with older patient age, practices outside of a major city, and patients who were long-time clients of a practice. Patients of larger primary care practices and were younger in age were less likely to have an identifier recorded.^31^

Since there is no universally accepted definition of Indigenous identity, there is no gold standard for how to ask service users about Indigenous identity or how to identify who is Indigenous. These complexities, nevertheless, do not justify excluding possibilities for Indigenous identifiers in administrative databases.^4^ Rather, organizations need to work in partnership with local Indigenous nations to ensure that any Indigenous identifiers appropriately reflect their definitions of nation membership.^20^ Health care organizations also need to be mindful about how they ask clientele about Indigenous identification because it can influence who is included and excluded. For example, a study examining maternal health across four sites found that the proportion of women who self-identified as Indigenous varied depending on the Indigenous identification criterion queried (i.e. cultural self-identification, language spoken or if they lived in an Indigenous household).^34^

### Data linkage

The other approach to identifying Indigenous people in health care records is the use of data linkage. Statistics Canada defines data linkage as ‘the process in which records or units from different data sources are joined together into a single file using nonunique identifiers, such as names, date of birth, addresses and other characteristics’^35^ (p. 1). Data linkage allows access to a range of information that could not have otherwise been retrieved from a single source.^36^

There are two approaches to exact matching data linkage: deterministic and probabilistic. Deterministic data linkage is the simplest form and produces linked records based on common identifiers or variables among the available data sources, such as birth date, health number or address.^35^ Probabilistic record linkage identifies the likelihood that two records are a match, based on the included identifiers or variables. Even if the records matched are not in complete agreement for each variable, they can be linked together to build a set of potential pairs.^35^ Probabilistic linkage is often more time-consuming and requires specialized software than deterministic linkage, but often produces more reliable results in comparison to deterministic data linkage.^35^

The International Group for Indigenous Health Measurement have argued that data linkage may be the most practical short-term corrective method to enhance Indigenous identity identification in administrative and vital statistics datasets.^19^ As information technologies have become more efficient, data linkage is becoming more effective and cost-efficient, as well as beneficial to the monitoring, measurement and evaluation of health care systems and health care outcomes.^37^ There is also a reduced burden on individuals to complete conventional surveys or self-disclose Indigenous identity in health care settings.

Although data linkage is regarded as less invasive, in comparison to collecting an identifier within health care settings, there are still ethical considerations to account for. Indigenous researchers and leaders have raised ethical considerations regarding data linkage, specifically when such linkages occur without Indigenous consent.^37^ For example, Indigenous researchers in Australia have raised concerns about the limited mechanisms available to ethically govern Indigenous data and data linkages in the country. They argue that, in Australia, there is a general lack of transparency regarding who makes decisions regarding the Indigenous health care data that is currently available and how it is used by researchers and the health care system.^38^

In a recent publication, a group of Indigenous health researchers noted that discussions about data linkage do not always recognise or address the sensitive nature of linking Indigenous population health data.^37^ They introduced the SEEDS principles, a living and expanding set of guiding principles for data linkage that: (1) prioritizes Indigenous Peoples’ right to Self-determination, (2) makes space for Indigenous Peoples to Exercise sovereignty, (3) adheres to Ethical protocols, (4) acknowledges and respects Data stewardship and governance and (5) works to Support reconciliation between Indigenous nations and settler states. They argue that data linkage without explicit consent is a violation of Indigenous peoples’ inherent rights.

First Nations across Canada have initiated First Nations-led data linkage efforts to access nation and region-specific health data.^39–41^ Typically, the Indian Registration System data is linked to administrative data sets held by provincial health or social departments. Data linkages can aid in rebuilding nations and exercising rights to self-determination. Furthermore, data obtained through linkages can assist with First Nations advocacy efforts to access resources to better address the social determinants of health.^40^ More recently, Inuit and Métis nations across Canada have also began using data linkage to identify health care priorities, disparities and mortality. A limitation is that the Indian Registration System is limited only to individuals who are registered under the Indian Act, which excludes 30% of the Indigenous population in Canada.^19^

### Indigenous data governance

Much of the academic and grey literature focused on the challenges in obtaining high-quality Indigenous health data, which focussed researchers’ attention on statistical issues – such as consistent Indigenous identifiers across administrative databases and sample size. However, more broadly, such discussions about improving availability of Indigenous health data also require concerted effort for considering the advancement of Indigenous data sovereignty through enhanced Indigenous data governance.^17^ Indigenous governance should be a key consideration for the entirety of the data lifecycle including conceptualization, collection, data linkage, ownership, analysis and dissemination. Statistical organizations, governments and health care service providers are called on to build meaningful and formal partnerships that recognise Indigenous sovereignty and self-determination with regards to their community data.^3,37^ Principles of Indigenous data sovereignty include that (1) Indigenous peoples having the power to determine who is counted among them and (2) data reflect the interests and priorities of Indigenous peoples and tribal communities, not only dictating the content of data collected about them but also retaining the power to determine who can access that data.^8^

There are several different frameworks and principles for Indigenous data sovereignty. For example, the CARE Principles refer to Collective benefit, Authority to control, Responsibility and Ethics.^42^ The First Nations Information Governance Centre outlines Ownership, Control, Access and Possession (OCAP®) principles are one example of Indigenous data governance principles.^43^ OCAP® principles were established in 1998 to ensure that First Nations own their information and are stewards of it, similar to the way they are stewards of their own lands. The principles of OCAP® also ensure that information is used in a way that maximizes the benefit for First Nations while minimizing potential for harm.^43^ Formal terms of reference between health care organizations, governments and Indigenous nations are encouraged to ensure that Indigenous nations are able to access data that pertains to them, can expect accurate data and contribute to the strengthening of data collection, analysis and interpretation.^2^

### Cultural appraisal findings

We analyzed 46 of the included academic articles using the QAT criteria, described in the Methods section (Table 3). Of the 46, only one article reported all 14 criteria.^7^

**Table 3.** QAT results.

QAT criteria^15^	Yes	Partial	No
1. Did the research respond to a need or priority determined by the community?	6	6	34
2. Was community consultation and engagement appropriately inclusive?	11	4	31
3. Did the research have indigenous research leadership?	4	7	35
4. Did the research have indigenous governance?	5	2	39
5. Were local community protocols respected and followed?	1	0	45
6. Did the researchers negotiate agreements in regard to rights of access to indigenous people's existing intellectual and cultural property?	1	1	44
7. Did the researchers negotiate agreements to protect indigenous people's ownership of intellectual and cultural property created through the research?	4	2	40
8. Did indigenous people and communities have control over the collection and management of research materials?	4	2	40
9. Was the research guided by an indigenous research paradigm?	2	1	43
10. Did the research take a strengths-based approach, acknowledging and moving beyond practices that have harmed indigenous peoples in the past?	2	5	39
11. Did the researchers plan and translate findings into sustainable changes in policy and/or practice?	14	9	23
12. Did the research benefit the participants and indigenous communities?	9	7	30
13. Did the research demonstrate capacity strengthening for indigenous individuals?	7	0	39
14. Did everyone involved in the research have opportunities to learn from each other?	7	1	38

Overall, the studies scored the highest on criterion 11, that is whether the researchers provided a plan to translate the findings into sustainable changes in policy or practice. However, Only half of the studies met the criterion either fully (*n* = 14) or partially (*n* = 9).

The second highest scorer was criterion 2, that is, engagement of diverse Indigenous communities (yes = 11, partial = 4).

Only 16 articles (yes = 9, partial = 7) met criterion 12, that is, produced meaningful benefits for the research participants and Indigenous communities more broadly.

The included studies performed lowest on criterion 5, acknowledging and following local Indigenous community protocols (no = 45), and criterion 6, whether the research team had agreements regarding Indigenous peoples’ existing intellectual and cultural property (no = 44).

Despite 11 studies demonstrating the engagement of Indigenous peoples within the study (criterion 2), only four of the included studies clearly identified Indigenous research leadership, which we defined as first, second or last author identifying as Indigenous within the article (criterion 4). It is important to note, however, that these results may reflect limitations in our research method, as discussed below.

## Discussion

This scoping review raises important considerations for improving Indigenous health data, a powerful tool for health care planning, surveillance and prevention. The lack of Indigenous specific health care data makes it difficult for Indigenous nations and health care services to develop population specific health care approaches. Both the academic and grey literature is consistent in arguing that health care organizations, health care providers and governments that do not collect Indigenous identity data ought to begin examining the possibilities in conjunction with Indigenous nations to address the data gap.

The history and ongoing legacy of colonialism has resulted in Indigenous peoples’ overall distrust of health care systems, research institutions and governments. In the spirit of reconciliation, non-Indigenous organizations must take meaningful steps towards addressing the Indigenous health gap in full partnership with Indigenous nations. Such partnerships will also ensure that research initiatives meet the specific data needs of Indigenous nations and peoples, as opposed to imposing non-Indigenous research agendas set by non-Indigenous governments and health care organizations.

We examined two potential ways to identify Indigenous peoples in administrative data. The first was voluntary self-identification of Indigenous identity in health care settings. Self-identification requires that health care settings provide a culturally safe environment to encourage self-disclosure of Indigenous identity. Indigenous identity disclosure must be voluntary and rely only on self-identification. If an Indigenous identifier is implemented, it is crucial that frontline staff receive adequate training on cultural safety in data collection. Furthermore, the identifier must be collected systematically to ensure the data is reliable and accurate. For example, despite Australia having an Indigenous identifier mandated by law, the identifier is still not systematically collected across health care sites and Indigenous people are frequently under-identified.^22,28^ Under-identification can also be attributed to the general distrust of health care settings among Indigenous people. Cultural safety within health care settings could be enhanced through health care partnerships with Indigenous organizations and nations, requiring a representative workforce in all positions in health care settings. For example, health services and government organizations could employ Indigenous statisticians and work in partnership with local Indigenous organizations to analyze Indigenous health data to ensure that analyses do not perpetuate harmful stereotypes.^12^

The other method of Indigenous identification is through data linkages in pre-existing administrative datasets. Data linkage is beneficial for several reasons. The method does not require individuals to self-disclose Indigenous identity, which allows for individual privacy. It is also a more cost-effective method of gathering data, especially for populations that are rural or remote.^44^ Data linkage can also identify individuals as Indigenous who would otherwise not be identified as such.^41,45,46^ Indigenous nations across Canada have demonstrated that Indigenous-led data linkage can aid in Indigenous sovereignty and self-government.^39–41,43^ While data linkage is a powerful and useful tool, there are also concerns about its use as well as challenges. Researchers in Australia have raised concerns that there are limited mechanisms to govern Indigenous data held by non-Indigenous governments and a general lack of transparency regarding data linking decisions.^38^ There is also the challenge of interoperability of data systems and platforms that exist outside of government such as primary health care data, disease registries and surveillance systems.^38^

We applied the QAT to all the research articles in the QAT, which revealed important findings about Indigenous research more generally. Overall, the QAT scores revealed that researchers often take steps to engage Indigenous peoples in the research process and offer sustainable policy level changes that can benefit Indigenous people. In this research, most of the articles offered policy-oriented solutions for improving Indigenous health data.

The QAT also revealed that there is still a lot of work to be done on improving Indigenous leadership and meaningful collaboration in research initiatives. Future research could include creating formal agreements that include acknowledging Indigenous peoples existing intellectual and cultural property as well as any data collected during the research. The QAT scores may also reflect a necessary shift in scientific reporting more generally. It is likely that some of the research teams did engage in more nuanced engagement with Indigenous peoples and nations as well as having Indigenous leadership in the research process, but were constrained by conventional scientific reporting standards that have historically not valued the reporting of Indigenous involvement or social location of authors. The use of QAT is beneficial for conducting systematic reviews and could also be used as guiding principles to consider in the early planning stages of research projects about Indigenous health.

## Limitations

There are two main limitations to this study, both concerning the QAT analysis. First, we found that the studies performed lowest on acknowledging and following local Indigenous community protocols and on whether the research team had agreements regarding Indigenous peoples’ existing intellectual and cultural property. It is possible that these studies did engage those criteria, they were not explicitly discussed within the articles and as such we could detect them.

Second, we found that only four of the studies clearly identified Indigenous research leadership in the research. It is possible that there were more Indigenous authors, but as we only relied on information contained in the articles, it is possible that we did not identify some Indigenous leading authors.

## Conclusion

This scoping review demonstrates that Indigenous health data is becoming a topic of global interest among health care organizations, researchers and Indigenous nations alike globally. Indigenous nations, organizations and researchers have advocated that, while access to accurate and reliable Indigenous health data is urgently needed, data collection, linkage and analysis should not occur without full collaboration with Indigenous peoples. The QAT demonstrated that most academic articles did not explicitly identify how Indigenous peoples were engaged in the research project. This finding calls for a larger culture shift in how research is reported, and such information ought to be included within the article for researchers to make assessments on the quality of the research from an Indigenous perspective.

## Supplemental Material

Supplemental Material - Indigenous identity identification in administrative health care data globally: A scoping review Supplemental Material for Indigenous identity identification in administrative health care data globally: A scoping review by Mandi Gray, Kienan Williams, Richard T. Oster, Grant Bruno, Annelies Cooper, Chyloe Healy, Rebecca Rich, Shayla Scott, Gary Teare, Samara Wessel, and Rita Henderson in Journal of Health Services Research & Policy

## References

[1] Madden R . Statistics on Indigenous peoples: international effort needed. Stat J IAOS 2016; 32: 37–41.

[2] Chino M Ring I Pulver LJ , et al. Improving health data for indigenous populations: the international group for indigenous health measurement. Stat J IAOS 2019; 35: 15–21.

[3] Steffler J . The indigenous data landscape in Canada: an overview. Aborig Policy Stud 2016; 5: 149–164.

[4] Madden R Coleman C Mashford-Pringle A , et al. Indigenous identification: past, present and a possible future. Stat J IAOS 2019; 35: 23–27.

[5] MMIWGS2S . Creating new pathways for data: the 2021 national action plan data strategy. https://mmiwg2splus-nationalactionplan.ca/wp-content/uploads/2021/06/The-2021-National-Action-Plan-Data-Strategy_EN.pdf (2021, accessed June 2022).

[6] Truth and Reconciliation Commission of Canada . Truth and reconciliation commission of Canada: Calls to action, https://ehprnh2mwo3.exactdn.com/wp-content/uploads/2021/01/Calls_to_Action_English2.pdf (2015, accessed June 2022).

[7] Smylie J Firestone M . Back to the basics: identifying and addressing underlying challenges in achieving high quality and relevant health statistics for Indigenous populations in Canada. Stat J IAOS 2015; 31: 67–87. 26793283 10.3233/SJI-150864 PMC4716822

[8] Walker J Lovett R Kukutai T , et al. Indigenous health data and the path to healing. Lancet 2017; 390: 2022–2023. 29115232 10.1016/S0140-6736(17)32755-1

[9] Hay T Fiddler TR . Inventing the thrifty gene: the science of settler colonialism. University of Manitoba Press, 2021.

[10] Mosby I . Administering colonial science: nutrition research and human biomedical experimentation in aboriginal communities and residential schools, 1942–1952. Histoire sociale/Social history 2013; 46: 145–172.

[11] McMillen C . Indigenous Peoples, tuberculosis research and changing ideas about race in the 1930s. CMAJ (Can Med Assoc J) 2021; 193: E1666–E1668. 34725117 10.1503/cmaj.210613 PMC8565975

[12] Walter M Andersen C . Indigenous statistics: a quantitative research methodology. Routledge, 2016, pp. 158.

[13] Arksey H O’Malley L . Scoping studies: towards a methodological framework. Int J Soc Res Methodol 2005; 8: 19–32.

[14] Levac D Colquhoun H O’Brien KK . Scoping studies: advancing the methodology. Implement Sci 2010; 5: 69–78. 20854677 10.1186/1748-5908-5-69 PMC2954944

[15] Harfield S Pearson O Morey K , et al. Assessing the quality of health research from an Indigenous perspective: the Aboriginal and Torres Strait Islander quality appraisal tool. BMC Med Res Methodol 2020; 20: 79–88. 32276606 10.1186/s12874-020-00959-3 PMC7147059

[16] Chambers LA Jackson R Worthington C , et al. Decolonizing scoping review methodologies for literature with, for, and by indigenous peoples and the African diaspora: dialoguing with the tensions. Qual Health Res 2018; 28: 175–188. 29182046 10.1177/1049732317743237

[17] Griffiths K Coleman C Al-Yaman F , et al. The identification of Aboriginal and Torres Strait Islander people in official statistics and other data: critical issues of international significance. Stat J IAOS 2019; 35: 91–106.

[18] Balestra C Fleischer L . Diversity statistics in the OECD: how do OECD countries collect data on ethnic, racial and indigenous identity? https://www.oecd-ilibrary.org/economics/diversity-statistics-in-the-oecd_89bae654-en (2018, accessed 26 Jul 2022).

[19] Coleman C Elias B Lee V , et al. International group for indigenous health measurement: recommendations for best practice for estimation of indigenous mortality. Stat J IAOS 2016; 32: 729–738.

[20] Gartner DR Wilbur RE McCoy ML . ‘American Indian’ as a racial category in public health: implications for communities and practice. Am J Public Health 2021; 111: 1969–1975. 34709855 10.2105/AJPH.2021.306465 PMC8630477

[21] Andersen C . The colonialism of Canada’s Métis health population dynamics: caught between bad data and no data at all. J Popul Res 2016; 33: 67–82.

[22] Gadsden T Wilson G Totterdell J , et al. Can a continuous quality improvement program create culturally safe emergency departments for Aboriginal people in Australia? A multiple baseline study. BMC Health Serv Res 2019; 19: 1–15. 30975155 10.1186/s12913-019-4049-6 PMC6458761

[23] Colmenares-Roa T Peláez-Ballestas I . Indigenous identification by health professionals in a Mexican hospital setting. Med Anthropol 2020; 39: 123–138. 31149848 10.1080/01459740.2019.1612394

[24] Lujan CC . American Indians and Alaska Natives count: the US Census Bureau’s efforts to enumerate the Native population. Am Indian Q 2014; 38: 319–341.

[25] Haozous EA Strickland CJ Palacios JF , et al. Blood politics, ethnic identity, and racial misclassification among American Indians and Alaska Natives. Journal of Environmental and Public Health 2014; 2014: 1–9. 10.1155/2014/321604 PMC3941118 24669226

[26] Briffa TG Sanfilippo FM Hobbs MST , et al. Under-ascertainment of Aboriginality in records of cardiovascular disease in hospital morbidity and mortality data in Western Australia: a record linkage study. BMC Med Res Methodol 2010; 10: 1–6. 21192809 10.1186/1471-2288-10-111 PMC3024993

[27] Schütze H Jackson Pulver L Harris M . What factors contribute to the continued low rates of Indigenous status identification in urban general practice? - a mixed-methods multiple site case study. BMC Health Serv Res 2017; 17: 1–12. 28143604 10.1186/s12913-017-2017-6 PMC5282656

[28] Ford BK Kong M Ward JS , et al. Incomplete recording of Indigenous identification status under-estimates the prevalence of Indigenous population attending Australian general practices: a cross sectional study. BMC Health Serv Res 2019; 19: 1–8. 31412854 10.1186/s12913-019-4393-6 PMC6693211

[29] Wynia MK Ivey SL Hasnain-Wynia R . Collection of data on patients’ race and ethnic group by physician practices. N Engl J Med 2010; 362: 846–850. 20200391 10.1056/NEJMsb0910799

[30] O’Loughlin M Harriss L Mills J , et al. Validating Indigenous status in a regional Queensland hospital emergency department dataset with patient-linked data. Med J Aust 2020; 212: 230–231. 31709545 10.5694/mja2.50401

[31] Thomson A Morgan S O’Mara P , et al. The recording of Aboriginal and Torres Strait Islander status in general practice clinical records: a cross-sectional study. Aust N Z J Public Health 2016; 40: S70–S74. 26123403 10.1111/1753-6405.12400

[32] Kehoe H . How can GPs drive software changes to improve healthcare for Aboriginal and Torres Strait Islander peoples? Aust Fam Physician 2017; 46: 249–253. 28376579

[33] de Witt A Cunningham FC Bailie R , et al. Identification of Australian aboriginal and Torres Strait Islander cancer patients in the primary health care setting. Front Public Health 2017; 5: 199. 28831386 10.3389/fpubh.2017.00199 PMC5549720

[34] Armenta-Paulino N Castello A Sandin Vazquez M , et al. How the choice of ethnic indicator influences ethnicity-based inequities in maternal health care in four Latin American countries: who is indigenous? Intern J Equity Health 2020; 19: 1–12. 10.1186/s12939-020-1136-6 PMC7069165 32164717

[35] Statistics Canada . 3.4.5 record linkage. https://www150.statcan.gc.ca/n1/edu/power-pouvoir/ch3/5214780-eng.htm (2021, accessed June 2022).

[36] Kisely S Pais J . Can administrative data provide insights into the mental health of Indigenous Queenslanders? Australas Psychiatr 2011; 19: S12–S16. 10.3109/10398562.2011.583047 21878008

[37] Rowe R Carroll SR Healy C , et al. SEEDS of indigenous population health data linkage. IJPDS 2021; 6: 1–10. 10.23889/ijpds.v6i1.1417 PMC8218891 34212119

[38] Ring I Griffiths K . Australian aboriginal and Torres Strait Islander health information: progress, pitfalls, and prospects. Int J Environ Res Publ Health 2021; 18: 10274. 10.3390/ijerph181910274 PMC8508232 34639572

[39] Tui’kn Partnership . Overview of the Nova Scotia Mi’kmaw client linkage registry. https://www.tuikn.ca/wp-content/uploads/2021/02/Overview-of-the-NSMCLR-Jan-2021.pdf (2021, accessed June 2022).

[40] Ramayanam VS Star L . Linking first Nations data to administrative health data within Manitoba. IJPDS 2018; 3.

[41] Jebamani LS Burchill CA Martens PJ . Using data linkage to identify first Nations Manitobans: technical, ethical, and political issues. Can J Public Health 2005; 96: S28–S32. 15686150 10.1007/BF03405313 PMC6976248

[42] Robinson CJ Kong T Coates R , et al. Caring for indigenous data to evaluate the benefits of indigenous environmental programs. Environ Manag 2021; 68: 160–169. 10.1007/s00267-021-01485-8 34046755

[43] First Nations Information Governance Centre . A First Nations data governance strategy: a response to direction received from First Nations leadership. https://fnigc.ca/wp-content/uploads/2020/09/FNIGC_FNDGS_report_EN_FINAL.pdf (2020, accessed June 2022).

[44] Lawrence D Christensen D Mitrou F , et al. Adjusting for under-identification of Aboriginal and/or Torres Strait Islander births in time series produced from birth records: using record linkage of survey data and administrative data sources. BMC Med Res Methodol 2012; 1: 1–13. 10.1186/1471-2288-12-90 PMC3493324 22747850

[45] Elias B Busby K Martens P . One little, too little: counting Canada’s indigenous people for improved health reporting. Soc Sci Med 2015; 138: 179–186. 26112164 10.1016/j.socscimed.2015.06.014

[46] Draper GK Somerford PJ Pilkington ASAG , et al. What is the impact of missing Indigenous status on mortality estimates? An assessment using record linkage in Western Australia. Aust N Z J Public Health 2009; 33: 325–331. 19689592 10.1111/j.1753-6405.2009.00403.x

