# Supplementary material for: Indigenous identity identification in administrative health care data globally: A scoping review
Source: J Health Serv Res Policy. 2023 Dec 15;29(3):210–21. doi: 10.1177/13558196231219955 (PMC11151709; doi:10.1177/13558196231219955)
Supplement: Supplemental Material - Indigenous identity identification in administrative health care data globally: A scoping review [file sj-pdf-1-hsr-10.1177_13558196231219955.pdf]

Online Supplement

S1: Academic articles included in the scoping review

1. Abouzeid, M., Bhopal, R. S., Dunbar, J. A., & Janus, E. D. (2014). The potential for measuring ethnicity and health in a multicultural milieu—The case of type 2 diabetes in Australia. *Ethnicity & Health*, *19*(4), 424–439. https://doi.org/10.1080/13557858.2013.828828

2. Andersen, C. (2016). The colonialism of Canada's Métis health population dynamics: Caught between bad data and no data at all. *Journal of Population Research*, *33*(1), 67–82.

3. Armenta-Paulino, N., Castello, A., Sandin Vazquez, M., & Bolumar, F. (2020). How the choice of ethnic indicator influences ethnicity-based inequities in maternal health care in four Latin American countries: Who is indigenous?. *International Journal for Equity in Health*, *19*(1), 31. https://doi.org/10.1186/s12939-020-1136-6

4. Baker, D. W., Cameron, K. A., Feinglass, J., Georgas, P., Foster, S., Pierce, D., Thompson, J. A., & Hasnain-Wynia, R. (2005). Patients' attitudes toward health care providers collecting information about their race and ethnicity. *Journal of General Internal Medicine*, *20*(10), 895–900. https://doi.org/10.1111/j.1525-1497.2005.0195.x

5. Baumeister, L., Marchi, K., Pearl, M., Williams, R., & Braveman, P. (2000). The validity of information on "race" and "Hispanic ethnicity" in California birth certificate data. *Health Services Research*, *35*(4), 869–883.

6. Bradshaw, P. J., Alfonso, H. S., Finn, J., Owen, J., & Thompson, P. L. (2009). Measuring the gap: Accuracy of the Western Australian hospital morbidity data in the identification of adult urban Aboriginal and Torres Strait Islander people. *Australian and New Zealand Journal of Public Health*, *33*(3), 276. ABI/INFORM Global; Nursing & Allied Health Premium; Politics Collection; Public Health Database; Publicly Available Content Database.

7. Briffa, T. G., Sanfilippo, F. M., Hobbs, M. S. T., Ridout, S. C., Katzenellenbogen, J. M., Thompson, P. L., & Thompson, S. C. (2010). Under-ascertainment of Aboriginality in records of cardiovascular disease in hospital morbidity and mortality data in Western Australia: A record linkage study. *BMC Medical Research Methodology*, *10*(100968545), 111. https://doi.org/10.1186/1471-2288-10-111

8. Callister, P., Didham, R., Potter, D., & Blakely, T. (2007). Measuring Ethnicity in New Zealand: Developing Tools for Health Outcomes Analysis. *Ethnicity & Health*, *12*(4), 299–320. https://doi.org/10.1080/13557850701300699

9. Carol Chiago Lujan. (2014). American Indians and Alaska Natives Count: The US Census Bureau's Efforts to Enumerate the Native Population. *American Indian Quarterly*, *38*(3), 319. https://doi.org/10.5250/amerindiquar.38.3.0319

10. Chino, M., Ring, I., Pulver, L. J., Waldon, J., & King, M. (2019). Improving health data for indigenous populations: The international group for indigenous health measurement. *Statistical Journal of the IAOS*, *35*(1), 15–21. https://doi.org/10.3233/SJI-180479

11. Christensen, D., Davis, G., Draper, G., Mitrou, F., McKeown, S., Lawrence, D., McAullay, D., Pearson, G., Rikkers, W., & Zubrick, S. R. (2014). Evidence for the use of an algorithm in resolving inconsistent and missing Indigenous status in administrative data collections. *Australian Journal of Social Issues*, *49*(4), 423–443. https://doi.org/10.1002/j.1839-4655.2014.tb00322.x

12. Clark, W., Lavoie, J.G., Nickel, N., & Dutton, R. (2020). Manitoba Inuit Association's rapid response to include an Inuit identifier within Manitoba COVID diagnostic tests. *American Indian Culture and Research Journal*, 44(3). https://doi.org/10.17953/aicrj.44.3.clark_etal

13. Coleman, C., Elias, B., Lee, V., Smylie, J., Waldon, J., Hodge, F. S., & Ring, I. (2016). International Group for Indigenous Health Measurement: Recommendations for best practice for estimation of Indigenous mortality. *Statistical Journal of the IAOS*, *32*(4), 729–738. https://doi.org/10.3233/SJI-161023

14. Colmenares-Roa, T., & Peláez-Ballestas, I. (2020). Indigenous Identification by Health Professionals in a Mexican Hospital Setting. *Medical Anthropology*, *39*(2), 123–138. https://doi.org/10.1080/01459740.2019.1612394

15. Cormack, D. (2007). Making Ethnicity Data Count. *Best Practices Journal*, *9*.

16. Diaz, A., Soerjomataram, I., Moore, S., Whop, L. J., Bray, F., Hoberg, H., & Garvey, G. (2020). Collection and Reporting of Indigenous Status Information in Cancer Registries Around the World. *JCO Global Oncology*, *6*, 133–142. https://doi.org/10.1200/JGO.19.00119

17. de Witt, A., Cunningham, F. C., Bailie, R., Bernardes, C. M., Matthews, V., Arley, B., Meiklejohn, J. A., Garvey, G., Adams, J., Martin, J. H., Walpole, E. T., Williamson, D., & Valery, P. C. (2017). Identification of Australian Aboriginal and Torres Strait Islander Cancer Patients in the Primary Health Care Setting. *Frontiers in Public Health*, *5*, 199. https://doi.org/10.3389/fpubh.2017.00199

18. Draper, G. K., Somerford, P. J., Pilkington, A. S. A. G., & Thompson, S. C. (2009). What is the impact of missing Indigenous status on mortality estimates? An assessment using record linkage in Western Australia. *Australian and New Zealand Journal of Public Health*, *33*(4), 325–331. https://doi.org/10.1111/j.1753-6405.2009.00403.x

19. Elias, B., Busby, K., & Martens, P. (2015). One little, too little: Counting Canada’s indigenous people for improved health reporting. *Social Science & Medicine (1982)*, *138*(ut9, 8303205), 179–186. https://doi.org/10.1016/j.socscimed.2015.06.014

20. Espey, D. K., Jim, M. A., Richards, T. B., Begay, C., Haverkamp, D., & Roberts, D. (2014). Methods for Improving the Quality and Completeness of Mortality Data for American Indians and Alaska Natives. *American Journal of Public Health*, *104*(S3), S286–S294. https://doi.org/10.2105/AJPH.2013.301716

21. Espey, D. K., Wiggins, C. L., Jim, M. A., Miller, B. A., Johnson, C. J., & Becker, T. M. (2008). Methods for improving cancer surveillance data in American Indian and Alaska Native populations. *Cancer*, *113*(S5), 1120–1130. https://doi.org/10.1002/cncr.23724

22. Firestone, M., Smylie, J., Maracle, S., Spiller, M., & O’Campo, P. (2014). Unmasking health determinants and health outcomes for urban First Nations using respondent-driven sampling. *BMJ Open*, *4*(7), e004978–e004978. https://doi.org/10.1136/bmjopen-2014-004978

23. Ford, B. K., Kong, M., Ward, J. S., Hocking, J. S., Fairley, C. K., Donovan, B., Lorch, R., Spark, S., Law, M., Kaldor, J., & Guy, R. (2019). Incomplete recording of Indigenous identification status under-estimates the prevalence of Indigenous population attending Australian general practices: A cross sectional study. *BMC Health Services Research*, *19*(1), 567. https://doi.org/10.1186/s12913-019-4393-6

24. Gadsden, T., Wilson, G., Totterdell, J., Willis, J., Gupta, A., Chong, A., Clarke, A., Winters, M., Donahue, K., Posenelli, S., Maher, L., Stewart, J., Gardiner, H., Passmore, E., Cashmore, A., & Milat, A. (2019). Can a continuous quality improvement program create culturally safe emergency departments for Aboriginal people in Australia? A multiple baseline study. *BMC Health Services Research*, *19*. ABI/INFORM Global; Nursing & Allied Health Premium; Publicly Available Content Database. https://doi.org/10.1186/s12913-019-4049-6

25. Gartner, D. R., Wilbur, R. E., & McCoy, M. L. (2021). “American Indian” as a Racial Category in Public Health: Implications for Communities and Practice. *American Journal of Public Health*, e1–e7. https://doi.org/10.2105/AJPH.2021.306465

26. Gialamas, A., Pilkington, R., Berry, J., Scalzi, D., Gibson, O., Brown, A., & Lynch, J. (2016). Identification of Aboriginal children using linked administrative data: Consequences for measuring inequalities. *Journal of Paediatrics and Child Health*, *52*(5), 534–540. https://doi.org/10.1111/jpc.13132

27. Gibberd, A., Simpson, J., & Eades, S. (2017). Using family relationships to improve consistency of identification of Aboriginal people in linked administrative data: IJPDS (2017) Issue 1, Vol 1:215 Proceedings of the IPDLN Conference (August 2016). *International Journal of Population Data Science*, *1*(1). https://doi.org/10.23889/ijpds.v1i1.235

28. Grafova, I. B., & Jarrin, O. F. (2021). Beyond Black and White: Mapping Misclassification of Medicare Beneficiaries Race and Ethnicity. *Medical Care Research and Review: MCRR*, *78*(5), 616–626. https://doi.org/10.1177/1077558720935733

29. Griffiths, K., Coleman, C., Al-Yaman, F., Cunningham, J., Garvey, G., Whop, L., Pulver, L. J., Ring, I., & Madden, R. (2019). The identification of Aboriginal and Torres Strait Islander people in official statistics and other data: Critical issues of international significance. *Statistical Journal of the IAOS*, *35*(1), 91–106. https://doi.org/10.3233/SJI-180491

30. Haozous, E. A., Strickland, C. J., Palacios, J. F., & Solomon, T. G. A. (2014). Blood Politics, Ethnic Identity, and Racial Misclassification among American Indians and Alaska Natives. *Journal of Environmental and Public Health*, *2014*, 1–9. https://doi.org/10.1155/2014/321604

31. Jamieson, L., Hedges, J., Peres, M. A., Guarnizo-Herreño, C. C., & Bastos, J. L. (2021). Challenges in identifying indigenous peoples in population oral health surveys: A commentary. *BMC Oral Health*, *21*(1), 216. https://doi.org/10.1186/s12903-021-01455-w

32. Jorgensen, S., Thorlby, R., Weinick, R. M., & Ayanian, J. Z. (2010). Responses of Massachusetts hospitals to a state mandate to collect race, ethnicity, and language data from patients: A qualitative study. *BMC Health Services Research*, *10*(1), 352. https://doi.org/10.1186/1472-6963-10-352

33. Jarrín, O. F., PhD, RN, Nyandege, A. N., PhD, Grafova, I. B., PhD, Dong, X., MD, MPH, & Lin, H., MD, PhD. (2020). Validity of Race and Ethnicity Codes in Medicare Administrative Data Compared with Gold-standard Self-reported Race Collected During Routine Home Health Care Visits. *Medical Care*, *58*(1). Public Health Database. https://doi.org/10.1097/MLR.0000000000001216

34. Jebamani, L. S., Burchill, C. A., & Martens, P. J. (2005). Using Data Linkage to Identify First Nations Manitobans: Technical, Ethical, and Political Issues. *Canadian Journal of Public Health = Revue Canadienne de Santé Publique*, *96*(Suppl 1), S28–S32. https://doi.org/10.1007/BF03405313

35. Jim, M. A., Arias, E., Seneca, D. S., Hoopes, M. J., Jim, C. C., Johnson, N. J., & Wiggins, C. L. (2014). Racial Misclassification of American Indians and Alaska Natives by Indian Health Service Contract Health Service Delivery Area. *American Journal of Public Health*, *104*(S3), S295–S302. https://doi.org/10.2105/AJPH.2014.301933

36. Johnson, J. C., Soliman, A. S., Tadgerson, D., Copeland, G. E., Seefeld, D. A., Pingatore, N. L., Haverkate, R., Banerjee, M., & Roubidoux, M. A. (2009). Tribal Linkage and Race Data Quality for American Indians in a State Cancer Registry. *American Journal of Preventive Medicine*, *36*(6), 549–554. Applied Social Sciences Index & Abstracts (ASSIA); Sociology Collection. https://doi.org/10.1016/j.amepre.2009.01.035

37. Kamaka, M. L., Watkins-Victorino, L., Lee, A., Freitas, S. M., Ramsey, K. W., Quint, J., Ku, T. L., Nishizaki, K., & Kaholokula, J. K. (2021). Addressing Native Hawaiian and Pacific Islander Data Deficiencies Through a Community-based Collaborative Response to the COVID-19 Pandemic. *Hawai'i Journal of Health & Social Welfare*, *80*(10 Suppl 2), 36–45.

38. Kehoe, H. (2017). How can GPs drive software changes to improve healthcare for Aboriginal and Torres Strait Islander peoples? *Australian Family Physician*, *46*(4), 249–253. https://doi.org/10.3316/informit.741609332807554

39. Kisely, S., & Pais, J. (2011). Can administrative data provide insights into the mental health of Indigenous Queenslanders? *Australasian Psychiatry*, *19*(Suppl 1), S12–S16. APA PsycInfo®. https://doi.org/10.3109/10398562.2011.583047

40. Lavoie, J.G., McDonell, L., Nickel, N., Clark, W., Anawak, C., Anawak, J., Brown, L., Clark, G., Evaluardjuk-Palmer, M., Ford., F., Dutton, R., Katz, A., & Wong, S. (2021). Understanding Manitoba Inuit's social programs utilization and needs: Methodological innovations. *The International Indigenous Policy Journal*, 12(4). https://doi.org/10.18584/iipj.2021.12.4.13690

41. Lavoie, J.G., Romanescu, Razvan, G., Katz, A., & Nickel, N., (2020) Modeling the impact of the COVID-19 pandemic on First Nations, Metis, and Inuit Communities: Some considerations. *International Indigenous Policy Journal*, 11(3). https://doi.org/10.18584/iipj.2020.11.3.10733

42. Lawrence, D., Christensen, D., Mitrou, F., Draper, G., Davis, G., McKeown, S., McAullay, D., Pearson, G., & Zubrick, S. R. (2012). Adjusting for under-identification of Aboriginal and/or Torres Strait Islander births in time series produced from birth records: Using record linkage of survey data and administrative data sources. *BMC Medical Research Methodology*, *12*(1), 90. https://doi.org/10.1186/1471-2288-12-90

43. Madden, R. (2016). Statistics on indigenous peoples: International effort needed. *Statistical Journal of the IAOS*, *32*, 37–41.

44. Mak, D. B., & Watkins, R. E. (2008). Improving the accuracy of Aboriginal and non-Aboriginal disease notification rates using data linkage. *BMC Health Services Research*, *8*(1), 118. https://doi.org/10.1186/1472-6963-8-118

45. McNamara, B. J., Jones, J., Shepherd, C. C., Gubhaju, L., Joshy, G., McAullay, D., Preen, D. B., Jorm, L., & Eades, S. J. (2020). Identifying young Aboriginal and Torres Strait Islander children in linked administrative data: A comparison of methods. *International Journal of Population Data Science*, *5*(1). https://doi.org/10.23889/ijpds.v5i1.1100

46. Minore B, Katt M, & Hill ME. (2009). Planning without facts: Ontario's Aboriginal health information challenge. *Journal of Agromedicine*, *14*(2), 90–96. CINAHL Complete. https://doi.org/10.1080/10599240902739802

47. Morales, D. (2019). The mysterious case of the disappearing Indians: Changes in self-identification as indigenous in the latest inter-census period in Bolivia. *LATIN AMERICAN AND CARIBBEAN ETHNIC STUDIES*, *14*(2), 151–170. https://doi.org/10.1080/17442222.2019.1612829

48. Nelson, M. A., Lim, K., Boyd, J., Cordery, D., Went, A., Meharg, D., Jackson-Pulver, L., Winch, S., & Taylor, L. K. (2020). Accuracy of reporting of Aboriginality on administrative health data collections using linked data in NSW, Australia. *BMC Medical Research Methodology*, *20*(1), 267. https://doi.org/10.1186/s12874-020-01152-2

49. Neville, S. E., Taylor, L. K., Moore, H., Madden, R., Ring, I., Lisa Jackson Pulver, & Tickle, L. (2011). Using linkage between hospital and ABS mortality data to enhance reporting of deaths among Aboriginal and Torres Strait Islander peoples. *Australian and New Zealand Journal of Public Health*, *35*(6), 543–548. ABI/INFORM Global; Nursing & Allied Health Premium; Politics Collection; Public Health Database; Publicly Available Content Database.

50. O'Loughlin, M., Harriss, L., Mills, J., Thompson, F., & McDermott, R. (2020). Validating Indigenous status in a regional Queensland hospital emergency department dataset with patient- linked data. *Medical Journal of Australia*, *212*(5), 230–231. https://doi.org/10.5694/mja2.50401

51. Palaniappan, L. P., Wong, E. C., Shin, J. J., Moreno, M. R., & Otero-Sabogal, R. (2009). Collecting Patient Race/Ethnicity and Primary Language Data in Ambulatory Care Settings: A Case Study in Methodology. *Health Services Research*, *44*(5p1), 1750–1761. https://doi.org/10.1111/j.1475-6773.2009.00992.x

52. Pettersen, T., & Brustad, M. (2013). Which Sami? Sami inclusion criteria in population-based studies of Sami health and living conditions in Norway—An exploratory study exemplified with data from the SAMINOR study. *INTERNATIONAL JOURNAL OF CIRCUMPOLAR HEALTH*, *72*, 1–11. https://doi.org/10.3402/ijch.v72i0.21813

53. Pulver LRJ, Bush A, & Ward J. (2003). Identification of Aboriginal and Torres Strait Islander women using an urban obstetric hospital. *Australian Health Review*, *26*(2), 19–25. CINAHL Complete.

54. Ramayanam, V. S., & Star, L. (2018). Linking First Nations data to administrative health data within Manitoba. *International Journal of Population Data Science*, *3*(4). https://doi.org/10.23889/ijpds.v3i4.983

55. Randall, D. A., Lujic, S., Leyland, A. H., & Jorm, L. R. (2013). Statistical methods to enhance reporting of Aboriginal Australians in routine hospital records using data linkage affect estimates of health disparities. *Australian and New Zealand Journal of Public Health*, *37*(5), 442–449. https://doi.org/10.1111/1753-6405.12114

56. Ring, I., & Griffiths, K. (2021). Australian Aboriginal and Torres Strait Islander Health Information: Progress, Pitfalls, and Prospects. *International Journal of Environmental Research and Public Health*, *18*(19), 10274. https://doi.org/10.3390/ijerph181910274

57. Rotondi, M. A., O'Campo, P., O'Brien, K., Firestone, M., Wolfe, S. H., Bourgeois, C., & Smylie, J. K. (2017). Our Health Counts Toronto: Using respondent-driven sampling to unmask census undercounts of an urban indigenous population in Toronto, Canada. *BMJ Open*, *7*(12), e018936. https://doi.org/10.1136/bmjopen-2017-018936

58. Rowe, S. L., & Cowie, B. C. (2016). Using data linkage to improve the completeness of Aboriginal and Torres Strait Islander status in communicable disease notifications in Victoria. *Australian and New Zealand Journal of Public Health*, *40*(2), 148–153. ABI/INFORM

Global; Nursing & Allied Health Premium; Politics Collection; Public Health Database; Publicly Available Content Database. https://doi.org/10.1111/1753-6405.12434

59. Rowe R, Carroll SR, Healy C, Rodriguez-Lonebear D, Walker JD. SEEDS of Indigenous Population Health Data Linkage. IJPDS. 2021 Jun 30 [cited 2022 Nov 9];6(1). https://ijpds.org/article/view/1417

60. Sarfati, D., Garvey, G., Robson, B., Moore, S., Cunningham, R., Withrow, D., Griffiths, K., Caron, N. R., & Bray, F. (2018). Measuring cancer in indigenous populations. *Annals of Epidemiology*, *28*(5), 335–342. https://doi.org/10.1016/j.annepidem.2018.02.005

61. Sarfati, D., & Robson, B. (2015). Equitable cancer control: better data needed for indigenous people. *Lancet Oncology*, *16*(15), 1442–1444. Nursing & Allied Health Premium; Public Health Database. https://doi.org/10.1016/S1470-2045(15)00295-8

62. Schütze, H., Pulver, L. J., & Harris, M. (2016). The uptake of Aboriginal and Torres Strait Islander health assessments fails to improve in some areas. *Australian Family Physician*, *45*(6), 415–420.

63. Schütze, H., Jackson Pulver, L., & Harris, M. (2017). What factors contribute to the continued low rates of Indigenous status identification in urban general practice? - A mixed-methods multiple site case study. *BMC Health Services Research*, *17*(1), 95. https://doi.org/10.1186/s12913-017-2017-6

64. Scotney, A., Guthrie, J. A., Lokuge, K., & Kelly, P. M. (2010). “Just ask!” Identifying as Indigenous in mainstream general practice settings: A consumer perspective. *Medical Journal of Australia*, *192*(10), 609–609. https://doi.org/10.5694/j.1326-5377.2010.tb03651.x

65. Smith, N., Iyer, R. L., Langer-Gould, A., Getahun, D. T., Strickland, D., Jacobsen, S. J., Chen, W., Derose, S. F., & Koebnick, C. (2010). Health plan administrative records versus birth certificate records: Quality of race and ethnicity information in children. *BMC Health Services Research*, *10*, 316. ABI/INFORM Global; Nursing & Allied Health Premium; Publicly Available Content Database. https://doi.org/10.1186/1472-6963-10-316

66. Smylie, J., & Firestone, M. (2015). Back to the basics: Identifying and addressing underlying challenges in achieving high quality and relevant health statistics for indigenous populations in Canada. *Statistical Journal of the IAOS*, *31*(1), 67–87.

67. Stepanikova, I. (2010). Applying a status perspective to racial/ethnic misclassification: Implications for health. In S. R. Thye & E. J. Lawler (Eds.), *Advances in Group Processes* (Vol. 27, pp. 159–183). Emerald Group Publishing Limited. https://doi.org/10.1108/S0882-6145(2010)0000027009

68. Steffler, J. (2016). The Indigenous Data Landscape in Canada: An Overview. *Aboriginal Policy Studies*, *5*(2). https://doi.org/10.5663/aps.v5i2.26992

69. Stehr-Green, P., Bettles, J., & Robertson, L. D. (2002). Effect of racial/ethnic misclassification of American Indians and Alaskan Natives on Washington State death certificates, 1989-1997. *American Journal of Public Health*, *92*(3), 443–444. ABI/INFORM Global; Nursing & Allied Health Premium; Politics Collection; Public Health Database.

70. Taylor, L. K., Bentley, J., Hunt, J., Madden, R., McKeown, S., Brandt, P., & Baker, D. (2012). Enhanced reporting of deaths among Aboriginal and Torres Strait Islander peoples using linked administrative health datasets. *BMC Medical Research Methodology*, *12*, 91. Publicly Available Content Database. https://doi.org/10.1186/1471-2288-12-91

71. Tervonen, H. E., Purdie, S., & Creighton, N. (2019). Using data linkage to enhance the reporting of cancer outcomes of Aboriginal and Torres Strait Islander people in NSW, Australia. *BMC Medical Research Methodology*, *19*(1), 245. https://doi.org/10.1186/s12874-019-0884-8

72. Thompson, S. C., Woods, J. A., & Katzenellenbogen, J. M. (2012). The quality of Indigenous identification in administrative health data in Australia: Insights from studies using data linkage. *BMC Medical Informatics and Decision Making*, *12*, 133. Publicly Available Content Database. https://doi.org/10.1186/1472-6947-12-133

73. Thomson, A., Morgan, S., O'Mara, P., Tapley, A., Henderson, K., van Driel, M., Oldmeadow, C., Ball, J., Scott, J., Spike, N., McArthur, L., & Magin, P. (2016). The recording of Aboriginal and Torres Strait Islander status in general practice clinical records: A cross-sectional study. *Australian and New Zealand Journal of Public Health*, *40*(S1), S70–S74. https://doi.org/10.1111/1753-6405.12400

74. Waldon, J. (2019). Identification of indigenous people in Aotearoa-New Zealand-Ngā mata o taku whenua. *Statistical Journal of the IAOS*, *35*(1), 107–118. https://doi.org/10.3233/SJI-180490

75. 18. Walker, J., Lovett, R., Kukutai, T., Jones, C., & Henry, D. (2017). Indigenous health data and the path to healing. *The Lancet*, *390*(10107), 2022–2023. https://doi.org/10.1016/S0140-6736(17)32755-1

76. Wynia, M. K., Ivey, S. L., & Hasnain-Wynia, R. (2010). Collection of Data on Patients' Race and Ethnic Group by Physician Practices. *New England Journal of Medicine*, *362*(9), 846–850. https://doi.org/10.1056/NEJMsb0910799

77. Xu, F., Sullivan, E. A., Madden, R. C., Black, D., & Jackson Pulver, L. R. (2012). Improvement of maternal Aboriginality in NSW birth data. *BMC Medical Research Methodology*, *12*, 8. https://doi.org/10.1186/1471-2288-12-8

S2: Academic articles categorized by theme

Theme 1: Policy recommendations

Description: Policy oriented papers and commentary

Citation	Method	Location	Key Findings	Data Governance
Andersen (2016)	Commentary	Canada	Health data for Métis people is poorly collected. The data that does exist relies on colonial logic and a misunderstanding of Métis identity. The lack of data makes it difficult to create meaningful health policy. **Argues that a census question that is more attentive to the complexities of Métis history is to link Métis self-identification to Métis organization attachment (i.e., Are you a member of the Métis Nation?)**	No discussion.

Chino et al (2019)	Commentary	International	The International Group for Indigenous Health Measurement (IGIHM) has pursued several activities to improve health measurement of Indigenous people globally. There is an ongoing challenge to measure Indigenous health due to the misidentification of Indigenous people on administrative and health records. **The major issue underlying this is a problem of identification – who is considered Indigenous and how is this documented in health data? The IGIHM is attempting to address these challenges through sharing of best practices, lobbying national statistical organizations, and reaching out to countries beyond the four founding members of the group.** This work is intended to increase the awareness within national statistical organizations of the need to improve data and measurement methods, to better describe and help improve the health status of their Indigenous populations.	IGIHM includes Indigenous stakeholders and researchers. The key element of the group development was to connect people and ideas to focus on the unifying principle of the group: **"The right of Indigenous peoples to count and be counted."** Terms of reference to ensure that Indigenous populations have rights to all their data, can expect accurate data and contribute strengthening data collection, analysis, and interpretation at all levels.
Clark et al (2020)	Commentary	Canada	An Inuit specific approach to discussing key issues relating to data, inequities, monitoring, and future research opportunities because of the COVID-19 pandemic. Historically, there have been no mechanism to identify Inuit in health systems, at the point of care and in administrative health data. Identification is essential to ensuring that universal coverage to response health services exists as well as the ability to monitor and evaluate health systems to identify gaps. The inclusion of an Inuit identifier to COVID-19 is an exceptional tool for public health officials and the ability for the Manitoba Inuit Association to access Inuit specific real time data.	Manitoba Health's administrative data has been made available to researchers for decades through the Manitoba Centre for Health Policy at the University of Manitoba, the most comprehensive data repository in Canada. Indigenous organizations govern and oversee the research that intends to use data specific to the populations they represent.

Coleman et al (2016)	Commentary	Aus., Brazil, US, NZ & Canada	This paper provides best practices for Indigenous mortality reporting based on recommendations from the International Group for Indigenous Health Measurement (IGIHM).<br>There is a need for statistical agencies globally to meaningfully engage with Indigenous communities. Historically enumeration practices excluded Indigenous people. Refers to the United Nations Permanent Forum on Indigenous Peoples seven criteria for the identification of Indigenous people.<br>**Canada does not record Indigenous identity on birth or death registration.**<br>**Best practice: Differentiate depending on the needs of the Indigenous peoples of each country. Stress that Indigenous identity needs to be more than just a "check box" based on blood quantum or genealogical proof but that identity is personal, social, emotional, political, and financial.**<br>IGIHM Recommendations:<br>1. There should be Indigenous identifiers on national census, birth and death certificates using the same definitions and nomenclature.<br>2. Self-identification; relatives should identify for death records.<br>3. Indigenous engagement at all stages of data collection, dissemination of results and principles of Indigenous community ownership and reporting needs to be established and maintained.<br>4. Partnerships need to be established between Indigenous people and statistical organizations to plan for sustainable data collection.<br>5. Where vital statistics have incomplete identification or registration, triangulation from a variety of sources may be considered.<br>6. Best practice linkage practices should be established to enhance vital statistics.<br>7. Back casting for estimation of health trends needs to be based on sound underpinning assumptions of mortality trends to avoid circularity.<br>8. Indirect methods can be used for calculation of life	Indigenous engagement at all stages of data collection, dissemination of results and principles of Indigenous community ownership and reporting needs to be established and maintained.<br>**Vital statistics are typically held by the state. Ownership models such as formal data use agreement can be used to give communities control over analysis and dissemination.**

Cormack (2007)	Commentary	Aotearoa-NZ	Best practice guidelines for Indigenous/ethnicity data collection in primary care settings:<br>1. Practitioners should always use the standard ethnicity question developed from the Census should be used. Consistent use will give researchers the ability to compare across datasets over time.<br>2. This should always be done by self-identification and not guessed based on appearance or name.<br>3. Ethnicity data should be recorded at the most detailed level and aggregating at the recording or input stage should be avoided.<br>4. Allow people to identify with more than one ethnic group.	No discussion.

Gartner (2021)	Commentary	USA	**When public health considers health/disease of Indigenous people, it is done using a racial lens.** Requires a more complex understanding of identity formation for Indigenous people, including political status within Native nations. Data collection must portray the diversity that exists across tribes and the inaccuracy of data must be explicitly acknowledged. A third party should never assign racial identity according to their own perception of the individual. **Potential solutions for commonly encountered issues in Indigenous aggregation:** 1. Data aggregation severely limits the local applicability of research but is unavoidable if no effort is made to obtain tribal affiliation of participants. 2. Data aggregation may raise ethical concerns if appropriate tribal approvals are not sought during the research process. 3. Data aggregation across tribes obscures important health disparities between and within tribes.	Data aggregation is a powerful tool but must be done following Indigenous community guidelines. **Every data identifier should be able to appropriately represent the community (i.e., Indigenous, tribal affiliation).**

Griffiths (2019)	Commentary	Australia	Components for the consideration in the contemporary reporting of Indigenous people in official statistics: human rights recognition (i.e., definitions, identity, and identification) and statistics (i.e., methodologies and limitations in enumeration and reporting). To date, the focus has been on statistical issues. Griffiths advocates for the use of a human rights framework ensure that all levels of government collect accurate and quality information. **The issues with the current data extend beyond simply a problem of collection, rather, a national discussion must occur about how Indigenous leadership can be involved in data governance, which will require a formal mechanism to ensure self-determination.**	Addressing the concerns about Indigenous health data must be done with Indigenous leadership in national data governance. Data is tied to self-determination aspirations of Indigenous people.
Haozous et al (2014)	Historical Analysis (Qualitative)	USA	Two central problems contribute to accuracy in AI/AN health data: (a) misclassification and (b) the complexity in defining AI/AN identity. Though data linkage can improve the data, administrative data is incomplete (i.e., tribal registries, IHS). **Researchers and policy makers must understand the complexity of AI/AN identity and social construction of race including the difference between race and ethnicity and the history of AI/AN identity.** Reasons related to disparities in health status data and racial misclassification are complex and related to several factors for the AI/AN population that include (a) the system of care, (b) methods used in calculating disease rates, (c) limitations in data tracking systems, (d) political processes that redefine tribal enrollment, (e) stereotyping, and (f) systems that do not collect data.	Recommends community involvement in inclusion criteria for identification.

Jamieson et al (2021)	Commentary	International	Rigorous and comprehensive measurement of oral health burden of Indigenous peoples is an ethical issue, but the survey instruments and sampling procedures are not sufficiently inclusive. The authors identify several methodological issues: suboptimal identification of Indigenous populations; numerator-denominator bias and statistical analytic considerations. **Recommendations include consistent Indigenous identifier in all countries with Indigenous populations such as Census data, health service data, and vital statistics.**	There is also a need to involve Indigenous people along with culturally appropriate data governance structures to be overseen by a body of Indigenous stakeholders.
Jorgensen et al (2010)	Evaluation (Mixed Methods)	USA	**Requiring the collection of race, ethnicity and language data can be an effective method to promote performance monitoring and quality improvement,** thereby setting the stage for federal standards and incentive programs to eliminate racial and ethnic disparities in the quality of health care. Information systems remain a barrier. **Implementation of state-wide data collection regulation for hospitals improved data collection practices with standardization, staff training. Increase in hospitals using data collected after legislation**.	No discussion.

Madden et al (2019)	Commentary	International	The methods used to identify Indigenous people have been imposed by colonizers. Historically, Indian Agents were the ones to determine who was First Nations in relation to the Indian Act which continues to shape data collection today. **There is no gold standard for Indigenous identification but that does not mean it should not be included in national censuses and in birth and death registrations.** There is an urgent need for national efforts to develops a standard process for Indigenous identification.	Historically Indigenous societies have been characterized and labelled by settler or colonizing groups. **There is an urgent need for Indigenous groups to apply their own identification principles.**
Minore, Kat & Hill (2009)	Qualitative	Canada	The capacity to document Indigenous health and service utilization varied significantly and depended upon existing provincial/territorial health data sets. The inability to identify Indigenous Ontarians in the provincial health information system poses a significant challenge for those charged with planning and delivering healthcare.<br>The authors Identified seven key themes: (1) Data gap (2) Absence of ethnic identifier within administrative databases (3) barriers to database linkage (4) cross-jurisdictional care (5) privacy and confidentiality concerns (6) access and governance issues and (7) technical or capacity requirements.	Recognition of OCAP ® and that Indigenous people have a collective entitlement to information about their communities, which must be recognized. Indigenous stakeholders must be engaged throughout the process and procedures must be in place to ensure that the information is of high quality and kept in strict confidence. Negotiation shall occur on a strict project-by-project basis to ensure that the information meets the needs of Indigenous communities, healthcare planners and providers.

Ring & Griffiths (2021)	Policy Review (Qualitative)	Australia	Despite significant developments in Indigenous health Information in Australia in the last 25 years, many challenges remain. Still uncertainty about the measure of life expectancy, which are unreliable due to the changes in patterns of identification. There are limited mechanisms to govern Indigenous data within government. **There is no available information about who is making decisions regarding linked data.**	Indigenous people must be partners in data collection and governance to aid with the design, collection, and use of information.
Smylie & Firestone (2015)	Commentary	Canada	There are current Indigenous data quality challenges including misclassification errors and non-response bias systematically contributing to the underestimation of inequities in health determinants, health status and healthcare access among Indigenous and non-Indigenous people in Canada. This problem requires the revision of core health data sources to include relevant, consistent, and inclusive Indigenous self-identification.	Changes to healthcare data collection must be done in collaboration with Indigenous peoples and governing organizations. Indigenous right to govern and manage their own knowledge and information systems is inherent to self-determination.
Sarfati & Robson (2015)	Commentary	Australia	To monitor cancer incidence among Indigenous populations, Indigenous status needs to be recorded accurately in cancer registries and relevant population denominator data must be used to estimate incidence using a similar process that linked census-cancer data are used when ethnic origin is collected. Most countries rely on an unlinked method, but Indigenous status is measured differently in cancer and census data resulting in a numerator-denominator bias that most likely underestimates incidence of cancer among Indigenous people. There are issues of inconsistent data collection of ethnicity data because of assumptions of clerical staff.	Engagement with Indigenous people.

Sarfati et al (2018)	Commentary	International	To better monitor cancer incidence among Indigenous populations, the authors recommend the enumeration of Indigenous peoples within the baseline population and a mechanism for accurately identifying Indigenous people within cancer and mortality data. Global cancer registries need to begin collection Indigenous status. **The authors suggest solutions that can be implemented to strengthen the visibility of indigenous peoples, including, acknowledgment of the central importance of full engagement of indigenous peoples with all data-related processes, encouraging the use of indigenous identifiers in national and regional data sets and mitigation and/or careful assessment of biases inherent in cancer surveillance methods for indigenous peoples.**	Engagement of Indigenous people.

Steffler (2016)	Commentary	Canada	Despite Canada’s relatively strong international leadership in terms of Indigenous data availability, significant data collection challenges remain domestically. Most of the data sources do not include an Indigenous identifier nor have sufficient Indigenous representation in the sampling methodology to produce reliable disaggregated estimates.	**There is a growing number of data sources being developed by Indigenous peoples for Indigenous peoples.** Issues of governance of Indigenous information will continue to be a key consideration in Indigenous data advancements or developments. There needs to be more work to build respectful and effective research relationships and establishing formal understandings of what research collaboration means to Indigenous peoples. **Collaboration with Indigenous partners is the new norm and is fundamental, necessary, and prerequisite to obtaining high quality, robust information for all users.**
Waldon (2019)	Commentary	Aotearoa-NZ	There are two key aspects to ensure the collection of consistent data and to minimize bias. One is to ensure consistent application of ethnicity and descent protocols and the other is that when descent data is collected with iwi affiliation, there is undercounting which results in a source of bias and poor-quality statistics.	There is a need to incorporate Māori perspectives applied to official statistics.

Theme 2: Indigenous health surveys, census, birth and mortality data collection

Note: Data governance column excluded for the remaining tables because most papers did not address it.

Walker (2017)	Commentary	Canada, New Zealand, Australia	There are two underlying roots to Canada's Indigenous health information challenges: (1) the lack of relevant, consistent, and inclusive Indigenous identity indicators in population health data sets and (2) the need for meaningful single registry of a sub-population of Indigenous people. The existing data deficiencies mask health inequality. Data can also demonstrate progress (i.e., improvement in smoking rates, vaccine coverage, cardiovascular deaths).	Article 24 of the United Nations Declaration on the Rights of Indigenous Peoples asserts the right to achieve the highest attainable health. Article 24 does not provide guidance on governance of data.<br>**Principles of data sovereignty must ensure that:**<br>(1) Indigenous peoples have the power to determine who should be counted among them.<br>(2) that data must reflect the interests and priorities of Indigenous peoples and<br>(3) tribal communities must not only dictate the content of data collected about them but also have the power to determine who has access to that data as described by Snipp (2016).

Citation	Type	Location	Indigenous identifier collection details	Key Findings
Armenta-Paulino et al., (2020)	Quantitative	Latin America	Demographic surveys in four countries: Mexico, Peru, Guatemala, and Bolivia to identify maternal health care: self-identification, spoken Indigenous language, or Indigenous household.	The Indigenous identification criteria can have an Impact on the measurement of inequalities in the coverage of maternal health. **Due to the complexity and diversity of Indigenous identity, it is not possible to provide a definitive direction on the best way to define Indigenous populations to measure inequalities.** The proportion of Indigenous women changes significantly according to the Indigenous identification criterion used. Women who self-identify as Indigenous higher than those who identified that they speak an Indigenous language. **From a human rights perspective, self-identification should be considered the most appropriate criterion which ensures a greater proportion of Indigenous women.**
Baumeister et al (2000)	Mixed Methods	USA	Matched birth certificates with face-to-face post-partum interviews at 16 hospitals	**Maternal racial/ethnic information on birth certificate appears to be a valid measure of self-identified race and Hispanic ethnicity for all groups other than Native Americans.** Native American sensitivity was only 54%. **There was more discrepancy for those who reported multi-ethnic/racial identities.**

Briffa et al (2010)	Quantitative	Australia	Cardiovascular disease in morbidity data	Indigenous status is under-reported, particularly when the patient has "no say" in what goes into their record. Conclude that combining Indigenous determinations in morbidity and official death records will increase ascertainment of unlinked morbidity. **The under-identification of Indigenous status is high in death records.**
Callister et al., (2007)	Quantitative	Aotearoa -NZ	2001 census	**Due to migration and marriage, there is greater need to move from single group-race-based measure towards culturally based complex ethnicity measures.** In 2001, 7.9% gave more than one response to the question. For Indigenous people, those who recorded Maori, only 56% solely reported Maori identity. **For younger populations, less than half of the Maori ethnic group reported only Maori identity.**
Chiago Lujan (2014)	Commentary	USA	US Census enumeration of American Indians and Alaska Natives	**It is important to have an accurate census count due to the implications for funding and representation.** The shift to self-identification can create problems and can result in under-counting. Challenges of data collection: population is mobile, transient, historical mistrust and other various methodological problems related to geographical challenges and language barriers.

Firestone et al., (2014)	Quantitative	Canada	Respondent driven sampling	Respondent Driven Sampling (RDS) is an effective sampling method in urban Aboriginal contexts as it builds on existing social networks and successfully identified a population-based cohort. The project developed a representative urban Aboriginal (First Nation) health database that provides quality health data.
Jim et al., (2014)	Quantitative	USA	Evaluated the racial misclassification of American Indians and Alaska Natives (AI/ANs) in cancer incidence and all-cause mortality data by Indian Health Service (IHS) Contract Health Service Delivery Area (CHSDA).	Sensitivity was significantly greater in Contract Health Service Delivery Area (CHSDA) counties. Classification ratios indicated less misclassification in CHSDA counties than non-CHSDA counties. Race misclassification was less in rural counties and in regions with the greatest concentrations of AI/AN persons (Alaska, Southwest, and Northern Plains). Limiting presentation and analysis to CHSDA counties helped mitigate the effects of race misclassification of AI/AN persons, although a portion of the population was excluded.
Morales (2019)	Quantitative	Bolivia	Self-identification in census-data	A notable decline in self-identification between the 2012 and 2001 census. The primary reason for the change is due to a change to the instrument for identifying Indigenous people, which is also tied to politics and socio-economic factors. In 2001, asked the respondent whether they feel part of one of the six Indigenous groups immediately presented (list) – 62% responded yes – easier to claim identification with Indigenous group – 2012 census asked – do you belong to one of the native-indigenous or Afro-Bolivian nations of Bolivia?

Rotondi et al., (2017)	Quantitative	Canada	Respondent driven sampling	Respondent driven sampling and Indigenous-led enumeration methods will have broad impacts for government and health policy to improve healthcare access and other “hard to reach” populations. Large scale respondent driven surveys of the urban Indigenous population show that the most recent Canadian census has underestimated the size of the Indigenous population in Toronto.
Petterson & Brustad (2013)	Quantitative	Norway	Population based health study	Established 1 geographically based and 3 individual based Sámi population by applying diverse Sámi inclusion criteria to data from 17 rural municipalities in Norway. Three principles (a) geographical location (b) linguistic connection (c) ethnic self-identification. The size and geography of the Sámi population were noticeably affected by what inclusion criteria were used.

Theme 3: Data linkage and algorithm development

Description: These studies include data linkage and the development of algorithms to identify Indigenous people in administrative data.

Citation	Type	Location	Data Linkage Method	Key Findings
Christensen (2014)	Quantitative	Australia	Combining administrative data on the same individuals from different sources using the Western Australian Data Linkage system using a range of algorithms.	The authors recommend the use of a multi-stage median algorithm for collecting and analyzing data on Indigenous identity. This algorithm incorporates kinship (mother or father ever identified un any dataset as Indigenous). The authors suggest that incorporating information collected, who reports/disclose identity, who collects it is also necessary for result accuracy.

Draper et al (2009)	Quantitative	Australia	Three sets of data: hospital morbidity database, mental health information system and midwives' notification system.	The authors used an algorithm to triangulate identity information. Better identification data collection significantly impacts mortality data. Most of the data collected is based on **self-identification, though Indigenous descent and community connection appear to be useful identifiers. The accuracy of the data relies on the opportunity to self-identify. Data linkage can improve existing information, but it is necessary to have systemic enhancement for data collection that can capture Indigenous identity across administrative data collections.**
Elias et al., (2015)	Mixed Methods	Canada	The authors linked the Indian Registration System (IRS) to track Indian status and eligibility with the Manitoba provincial health registry.	A legal review can assist to identify those who would have historically been excluded from the IRS. The legal analysis will help building a more representative ethno-cultural identifier for data linkage and disaggregation purposes. Although the IRS is a colonial tool, it has the greatest data linkage potential.
Espey et al (2008)	Quantitative	USA	Data linkage: Identified in medical record (presumed self-ID) or have ancestry in a federally recognized tribe to receive Indian Health Service services.	The classification of race for AI/AN cases in cancer registries can be improved by linking records to the IHS and stratifying by CHSDA counties. In total, 12,103 AI/AN individuals who had been classified as non-AI/AN were identified as AI/AN by the IHS linkage in these 49 states, ranging from 85 individuals in the Alaska region (3.4%) to 5297 individuals in the Southern Plains region (44.5%).
Espey et al (2014)	Quantitative	USA	Data linkage of US National Death Index records with Indian Health Service registration records to identify AI/AN death misclassification. Regional comparative analysis.	Data linkage is a useful but imperfect tool. Improving race classification over American Indian/Alaska Native (AI/AN) descent strengthens mortality data. Analyzing by geographic region can assist in efforts to reduce health disparities in AI/AN population. Misclassification ranged from 6.3% to 35.6%. The death rate remained 46% greater for AI/AN persons than for white persons.

Gialamas (2016)	Quantitative	Australia	Data linkage of Aboriginal ethnicity data linked to datasets recording development outcomes (literacy, numeracy, early development, census, and perinatal statistics).	Linkage of multiple datasets increases Aboriginal children identified as Aboriginal; different algorithms result in different estimates of inequalities. Linking the perinatal, births and the Australian Early Development Census or school enrolment dataset to create an 'ever-identified' algorithm provided a higher proportion of children identified as Aboriginal (3.4% and 4.7% identified as Aboriginal for the Australian Early Development Census and NAPLAN samples, respectively) than recorded in any single dataset.
Gibberd et al (2017)	Quantitative	Australia	Comparison of 3 algorithms used commonly to identify Aboriginal status via administrative data sets (birth records and registrations, hospital records, and birth defects). Algorithm performance tested using family relationships.	When many data sources are used, the most inclusive algorithm for identifying Aboriginal people (''ever-Aboriginal'') performed worse in assigning consistent Aboriginal status to family members than more specific algorithms. Use of the ever-Aboriginal algorithm may lead to underestimates of poor health outcomes in the Aboriginal population. People who could not be categorized as either Aboriginal or non-Aboriginal based on their own records could generally be categorized based on their relatives' Aboriginal statuses. Representation of some groups with few administrative records was improved by using relatives' records, including healthy people with no hospital records and stillborn infants.
Jebamani et al., 2005)	Quantitative	Canada	The Indian Registry Verification System was linked with the Manitoba Health Registry.	The linkage resulted in the identification of a substantial number of First Nations Manitoban's who would have otherwise not been counted by the Manitoba Health Registry which allowed for a more accurate picture of FN health status and use of health care services.

Johnson et al., (2009)	Quantitative	USA	State cancer registry – linkage to tribal membership registry	Cancer records tend to misclassify / ignore culture or identity aspects. The authors argue that the use of new technologies can enhance data such as probabilistic software. Authors used data linkage between tribal and state cancer registry data sets to improve racial classification validity and conclude that assessing tribal linkages is a simple, noninvasive way to improve the accuracy of state cancer data for AI/AN populations and to generate tribe-specific cancer information.
Kisely & Pais (2011)	Commentary	Australia	Review of the potential for data linkage studies for mental health of Indigenous Queenslanders	Indigenous identity in hospital morbidity, mental health and mortality is correct in 89% of the cases. Administrative data provides researchers and decision makers with accessible, cost-effective information without the intrusion of additional data collection. This can be a useful method in studying regional, rural, and remote populations where access may be difficult.
Lawrence et al., (2012)	Quantitative	Australia	Compared birth registration and the Western Australia Midwives System to compare consistency of determining Indigenous children	The authors found that record linkage of survey data to administrative data is useful to validate the quality of recording of demographic information. The identifier was not randomly distributed. **The births of children being identified as Indigenous only in the survey and not the administrative database was more likely to live in urban areas, disadvantaged communities and have only one parent who identified as Indigenous (usually the father).** These children were also more likely to have better health and wellbeing outcomes. **A major challenge in collecting this data is that while it is of interest to researchers, it is not tied to the direct needs of the agency providing services and may not always be rigorously pursued or collected. In many clinical spaces, there is a hierarchy of data collection.**

Lavoie et al., (2021)	Quantitative	Canada	Identifying all Inuit using their Nunavut Health Care Number card and Statistics Canada Kivalliq populations for the denominator, inferring population figures in between census years.<br>Probabilistic data linkage was also used by linking administrative data.	Manitoba, Canada does not have an Inuit identifier. Initially hoped to use administrative data from the Manitoba Population Research Data Repository from 1984-2016 but because of the provision of services to Nunavut Inuit changed jurisdictional responsibility 3 times during this period. Two interrelated approaches were used to identify Inuit in the health administrative datasets.
Lavoie et al. (2020)	Quantitative	Canada	Modeling of COVID-19 for First Nations, Métis and Inuit peoples in Manitoba, Canada.	Using modeling data requires access to expertise in programming and requires substantial computational resources that many Indigenous organizations and nations do not have access to due to insufficient resources and infrastructure.<br>Identify challenges modeling COVID-19 in FNMI populations: little dependable demographic data exists for FN not eligible for registration under the *Indian Act*, Inuit outside of Nunangat, and Metis; applicability of severe and critical case ratio to FNMI is unknown but is generally assumed that all three will be at higher risk of severe disease.<br>Opportunities/Potential sources of data: Population data for FN with status under the *Indian Act* is kept up to date by Indigenous Services Canada; Statistics Canada and the government of Quebec, Newfoundland and Labrador, Nunavut, and NWT publish population reports; epidemiological studies are produced by all provinces and territories as well as by research community.<br>Overall, modeling the impact of COVID-19 in FNMI populations is complicated. **Modeling the impact of COVID-19 on the urban Indigenous population is virtually impossible because dependable population estimates do not exist.**<br>**Indigenous communities and organization require accurate data to support pandemic plans.**

Mak & Watkins (2008)	Quantitative	Australia	Data linkage to improve accuracy of estimated notification rates for Sexually Transmitted Infections and blood borne viruses.	**Incompleteness of Indigenous data contributes to the over-estimation of risk associated with Indigenous identity for sexually transmitted infections and blood borne viruses.** Data linkage can be effectively used to improve the accuracy of estimated disease notification rates. **Using data linkage, the missing Indigenous status data was reduced by 74%.**
McNamara et al., (2020)	Quantitative	Australia	Linked administrative health data for children born (2000-2013): Indigenous identifier, Indigenous status of children, their parents, and/or grandparents, Indigenous status of mother or father on any of the child records.	**The Indigenous identifier of the child, parents, or grandparents established the largest cohort.** Additional children were identified by the other methods were from less disadvantaged and more urban areas and had better perinatal outcomes. **The demographic and perinatal health characteristics differed by status identification method.** Using perinatal records of the Indigenous identifier of parents and grandparents appeared to be more inclusive method to identifying young Indigenous children.
Nelson et al., (2020)	Quantitative	Australia	Linked surveys with administrative health data to assess accuracy.	A substantial number of Indigenous people are not reported as such in any of their records. There needs to be improvements made in reporting. **Record linkage should not replace efforts to improve recording of Indigenous people at the point of data collection and there is a need to address the barriers for self-identification**.
Neville et al (2011)	Quantitative	Australia	Development of 6 algorithms to enumerate deaths.	Data linkage can be used to improve the reporting of Indigenous people on routinely collected mortality data in Australia, New Zealand, and Canada.

Randall et al., (2013)	Quantitative	Australia	Admitted hospital patient data linked with mortality data (enhancement algorithm)	The aim of this study was not to identify individuals as Indigenous - but to use statistical means to explore the sensitivity of estimates of health disadvantage for Indigenous people to the way Indigenous status is determined and reported using linked hospital data. **Estimates of Indigenous health disparity can change depending on how Indigenous status is reported**. Recommend sensitivity analyses using several algorithms.
Ramayanam & Star (2018)	Quantitative	Canada	Advocate for data linkage to enhance First Nations data governance	Dataset linkage will help strengthen First Nations data governance in re-building nations and recognizing First Nations right to self-determination. Data linkage will provide meaningful data to advocate for First Nations rights to access resources and social determinants of health needed to achieve health equity in Manitoba. This dataset can only be accessed with an application to and approved by Manitoba First Nations Health Information Research Governance Committee.
Rowe & Cowie (2016)	Quantitative	Australia	Linked notifications with hospitalization data.	In the original dataset, Indigenous status was unknown for most of the notifications but could have been ascribed with data linkage. The data linkage led to substantial increase in notification rates for Indigenous people. **There is a range of methods available for linkage: "ever identified" methodology is the most used and most simple.**
Rowe et al., (2021)	Commentary	International	Indigenous led principles for data linkage of Indigenous population health data	The authors identify the SEEDS principles for Indigenous population health data linkages: 1) prioritize Indigenous Peoples' right to self-determination 2) make space for Indigenous Peoples to exercise sovereignty 3) adhere to ethical protocols 4) acknowledges and respects data stewardship and governance and 5) works to support reconciliation between Indigenous nations and settler states.

Stehr-Green et al., (2002)	Quantitative	USA	Developed an algorithm using record linkage of the Northwest Tribal Registry.	Improving identification of race/ethnicity from an authoritative external data source has the potential to greatly reduce the problem of misclassification. Methods to reduce misclassification can improve data quality and enhance efforts to measure and reduce racial/ethnic health disparities. **The authors identified matches for 2819 decedents, including 2405 (85.3%) who had been identified as American Indians and Alaskan natives in both data sets and 414 (14.7%) who had been misclassified as non–American Indians/ Alaskan Natives on the death certificates. A strong, statistically significant inverse association was found between blood quantum and misclassification: persons with a 100% blood quantum were less than one tenth as likely to be misclassified (4.0%) as persons with a less than 25% blood quantum (43.6%).**
Taylor (2012)	Quantitative	Australia	Data linkage – death registration and four population health datasets (hospitalization, emergency department attendances and birth)	Record linkage creates a statistical construct that helps to correct under-reporting of deaths and potential bias in mortality statistics for Indigenous peoples.
Thompson et al (2012)	Commentary	Australia	Administrative health data	Under identification of Indigenous status can be diminished through data linkage. **The under-identification of Indigenous status has different impacts in different disease contexts, generally resulting in the under-estimation of absolute and relative Indigenous health indicators but may also over-estimate Indigenous rates and differentials in the setting of stigma associated conditions such as sexually transmitted and blood-borne virus infections.** The under enumeration in the census also needs consideration.

Theme 4: Indigenous identity collection in health care settings

Xu et al. (2012)	Quantitative	Australia	Linked birth data from the Midwives Data Collection and the Registry of Births and Deaths	Linking birth data collections is feasible and will improve the statistical ascertainment of Aboriginal women giving birth in NSW. This will assist with targeting appropriate services in pregnancy and early childhood.

**Citation**	**Type**	**Location**	**Indigenous Identifier Collection Details**	**Key Findings**
Abouzeid et al (2014)	Qualitative	Australia	Standardized collection in electronic medical records	A review of 32 type 2 diabetes databases found that Indigenous status was recorded in 25 of the databases. **This was collected in two primary ways: a distinct question asking about Indigenous identity or as a response option in race/ethnicity questions.** The authors argue for a standardized approach to collecting self-identified ethnicity data.
Baker et al., (2005)	Quantitative	USA	Patient attitudes towards collecting race data among internal medicine patients	Cross-sectional survey of 220 general internal medicine patients. 80% of patients somewhat or strongly agreed that health care providers should collect info on race/ethnicity. 28% had significant discomfort, 58% were somewhat or very concerned that this information could be used to discriminate against patients.<br>*Note: this study only included 1 participant who identified as Native American

Bradshaw et al, (2009)	Mixed Methods	Australia	Hospital administrative morbidity data (urban population)	The sensitivity of coding Indigenous status in hospital admission significantly improved over time, exceeding 0.9 in every year since 2002. As a result, the Western Australia Hospital Morbidity Database has become a more accurate tool for the identification of adult Indigenous people. Accurate recording in more than 90% of hospital admissions. **There is no clear explanation as to what caused the improvement but may be due to initial hesitancy of clerical admissions to ask patients about their status and the increasing awareness of the value of identification.**
Colmenare-Roa & Peláez-Ballestas (2020)	Qualitative	Mexico	Hospital ethnography of Indigenous identity collection	Health service providers rarely consider Indigenous identity as relevant. **When service providers do take Indigenous identity into consideration, they base their identification on stereotypes of Indigenous people based on language, place of origin, cultural practices, and poverty.** Most service providers did not document Indigenous identity and patients did not disclose due to fear of discrimination. Few service providers used translator services or other resources for their Indigenous patients. Hospitals should identify Indigenous patients to promote medical and social practices. Identification must avoid stereotypical connections between ethnic identity, poverty, and vulnerability.

Diaz et al (2020)	Qualitative	International	Global survey of Indigenous identifier collection in population-based cancer registries.	Total of 83 population-based cancer registries from 25 different countries were included, total of 66% reported collecting Indigenous identifier. The quality of the variable was assessed in only 38% of these and accuracy in 47%. **The key barriers to data collection: lack of data collection at the point of care, lack of transfer of Indigenous status to the cancer registry, inadequate information systems and legislative limitations.**
de Witt (2017)	Qualitative	Australia	Identify Indigenous cancer patients in primary health centres.	An examination of ten primary health care centers, all of which reported the ability to identify Indigenous cancer patients attending their services. Among these centers, there were six different Patient Care Information Systems (PCIS) to manage the records, all of which recorded Indigenous status differently. **Recommendation: It is crucial to be able to easily identify Indigenous cancer patients in health services in the primary health care setting to monitor progress, improve and evaluate care.** It is also necessary for primary health care center staff to receive training and support to use the PCIS. The authors identified a knowledge gap between staff members and between primary care centers in their ability to use the PCIS.

Ford et al., (2019)	Qualitative	Australia	Examination of data collection in primary care.	Almost half of the patient records had missing data, either incomplete, under-reported or completely missing. **Most clinicians admitted that they did not collect the data systematically**. Relying solely on self-disclosure will miss information because individuals do not always disclose their identity. **The author recommends linking different datasets and shifting organizational culture to encourage data collection for improved health outcome.**
Gadsden et al (2019)	Evaluation	Australia	Emergency department	This study assessed the impact of an emergency department quality improvement program on the accuracy of recording Indigenous status in ED information systems, incomplete ED visits among Indigenous patients and cultural appropriateness of ED systems and environments. The accurate recording of Indigenous status varied among the participating emergency departments ranging from 45.5% to 87.2% with an average of 76% (n=8). In two of the ED the quality improvement program was associated with an increase in the accurate recording of Indigenous patients. **Indigenous patients will not identify as Indigenous if they perceive the environment is culturally unsafe and there is missing data on Aboriginal experiences in the ED due to this.**

Grafova & Jarrin (2020)	Quantitative	USA	Medicare beneficiaries – home and healthcare services	The authors examined differences between states race/ethnicity data. **They found substantial variation between states in Medicare administrative data misclassification of self-identified American Indian/Alaska Native beneficiaries.** Caution should be used when interpreting state level health care disparities and minority health outcomes based on existing variables in Medicare data sets. **The use of self-reported race/ethnicity data may be used to improve the accuracy of minority health and health disparities reporting and research.**
Jarrín et al (2020)	Quantitative	USA	Medicare beneficiaries – home and healthcare services	Examined the misclassification of Medicare beneficiaries' race/ethnicity in administrative data sources. Compared the validity of two race/ethnicity variables in Medicare administrative data (EDB) and Research Triangle Institute (RTI) against self-reported race/ethnicity. **The EDB and RTI race variable have high validity for Black patients and low validity for American Indian/Alaskan Native. The data for American Indians/Alaskan Natives is often incomplete or inaccurate, which limits the understanding of disparities in healthcare access, quality, and outcomes.**
Kamaka et al (2021)	Commentary	USA	Co-created community-led initiative of self-disclosure of Native Hawaiian and Pacific Islander identity at COVID testing sites.	There is a need for accurate racial/ethnic data collection at COVID test sites. Disaggregated racial/ethnic data is paramount to understanding and developing effective policies and health inequities. Such data collection is based on collaborative work that honours culture and ensures Native Hawaiian, Pacific Islander and other minority populations are accounted for.

Kehoe (2017)	Commentary	Australia	Collection in primary care electronic medical record systems.	**There are several technical barriers for primary care providers to collect Indigenous identity data. It is argued that software providers make necessary changes to better capture the data.** This would be beneficial at numerous levels. For general practitioners this would mean better clinical care and billing. For patients this would result in improved access to preventative care, support services and more affordable medicine. At the population level, better data would improve service delivery.
O'Loughlin et al (2020)	Commentary	Australia	Compared face-to-face surveys with electronic medical record data in emergency department.	**The research found that it was more common for under-identification of Indigenous status rather than overidentification.** Sensitivity (self-identifying Indigenous people recorded by EDIS as being Indigenous) was 85.7%, specificity was 98.6% and overall accuracy was 97%.
Palaniappan et al., (2009)	Qualitative	USA	Collecting race/ethnicity and language data in ambulatory care.	Comparison of (1) mail vs. telephone vs. clinic visit questionnaire and (2) compare front desk method vs. exam room. Collecting data during clinic visits had the highest response rate. **The front desk method yielded higher response rate than in the exam room. This labour would require one FTE for data entry.** Patients were accommodating in providing the information with a high response rate.
Pulver et al., (2003)	Qualitative	Australia	Examined self-identification by survey vs. administrative data at obstetric hospital.	Indigenous people giving birth at an urban obstetric hospital are under-represented in official statistics. Authors recommend developing sensitive but specific questions to allow patients to disclose their status. This study found that the participants did not mind being asked about Indigenous identity.

Schutze et al (2016)	Qualitative	Australia	Self-identification in primary care (3 practitioners)	The biggest barriers to uptake in general practice, which remains below 22%, is due to low rates of Indigenous status identification and a lack of knowledge about it by practitioners.
Schutze et al (2017)	Qualitative	Australia	Self-identification in primary care	Indigenous people remain under-identified in general practice. **Several reasons are identified including: the lack of staff recognition that a problem exists, practice environments that do not promote identification, entrenched attitudes among staff and software limitations**. The authors advocate for a policy change mandating Indigenous identification and recording in general practice.
Scotney et al., (2010)	Commentary	Australia	Self-identification in primary care	Argue that physicians should “just ask” about Indigenous identity to ensure that the identifier question and explanation are conveyed consistently and appropriately. There are several themes that would influence decision to identify: previous racism; patient-doctor relationship; perception discussing identity would lengthen consultation times; assumed motives for asking; recognition of the culture and diversity of Indigenous Australians. **The principal message was that the process for asking needs to be brief and simple.**

Smith et al., (2010)	Quantitative	USA	Health plan administrative records vs. birth certificate records	Misclassification was most common in minority groups. **Positive predictive value was 1.2% for American Indians/Alaskan Native in comparison to 89.3% for white and 86.6% for Black children.** Racial and ethnic information improved with increasing number of medical visits. **The use of health plan administrative records alone leads to frequent misclassification for minority groups (i.e., Indigenous children) and those with multiple racial/ethnic identities.**
Stepanikova (2016)	Quantitative	USA	Examines differences between self-identified race/ethnicity and perceived race/ethnicity	The study did not focus on identity measurement, rather, demonstrates that perceived racism is independently linked to both measurements of identity (perceived vs. self-identification). Perceived attributed race/ ethnicity captures an aspect of racial/ethnic identity that is correlated, but not interchangeable, with self-identified race/ethnicity and contributes uniquely to perceived discrimination in health care. Applying the concept of multidimensionality of race/ethnicity to health disparities research may reveal understudied mechanisms linking race/ethnicity to health risks. **Found large discord between racial misclassification between groups (highest in Native Americans at 47%).**
Tervonen et al (2019)	Quantitative	Australia	Cancer registry using “ever reported” “reported on most recent record” “weight of evidence” and “multi-stage median”	**Most cancer cases with a status change after data linkage enhancement were originally recorded as non-Indigenous.** This was more common for males, people aged 25-34, people with cancer of unknown or localized spread, people in urban areas and areas with less socio-economic disadvantage.

Thomson et al (2016)	Quantitative	Australia	General practice clinical records	Nearly 20% of Indigenous patients did not have their status recorded in their clinical record indicating that recording may not be systematic. **Higher rates of identifier recording were associated with older patient agent; practice outside of a major city; patients who were not new to the practice; and the patient identifying as Indigenous. Patients of larger practices were less likely to have an identifier recorded**. Those who were younger were less likely to have an identifier recorded. **Identifier was not systematically collected but was when a condition associated with Indigeneity developed, such as diabetes.** Recommend that clinic staff have a systematic approach to collecting Indigenous identifier to inform policy and practice.
Wynia et al (2010)	Commentary	USA	Physician data collection practices	Reliable collection by physicians is uncommon. This is due to: **lack of financial support for data collection, privacy concerns, legal barriers, patient discomfort and that they saw no benefit of the data and felt it was not clinically relevant.** Physicians felt that racial profiling patients was harmful, and that race should not be used to infer information about health.

S3: Grey literature included in the scoping review

1. Alberta Health Services (2018) *Indigenous health transformational roadmap*. https://www.albertahealthservices.ca/assets/about/scn/ahs-scn-ppih-ih-roadmap.pdf
2. Australian Bureau of Statistics. (2012). *Perspectives on Aboriginal and Torres Strait Islander identification in selected data collection contexts* https://www.ausstats.abs.gov.au/ausstats/subscriber.nsf/0/04B81E4FA4BEA3EDCA257B04000FD8E6/$File/47260_2012.pdf
3. Australian Government (2020) *Voluntary Indigenous identifier (VII) framework: A framework for the collection, release, use and publication of VII data*. https://consultations.health.gov.au/indigenous-health/vii-framework/supporting_documents/VII%20Framework_CONSULTATION%20DRAFT_November%202020.pdf
4. Australian Government Department of Health and Ageing (2004) *Improving Indigenous identification in communicable disease reporting systems*. https://phidu.torrens.edu.au/pdf/1999-2004/improving_indigenous_reporting_2004.pdf
5. Australian Health Minister's Advisory Council (AHMAC). *National Aboriginal and Torres Strait Islander Health Data Principles*. https://www.aihw.gov.au/about-us/international-collaboration/international-group-indigenous-health-measurement
6. Australian Institute of Health and Welfare (2005). *International group for Indigenous health measurement*. https://www.aihw.gov.au/getmedia/125747f0-00ba-4183-bfc6-a89a21a425db/igihmv05.pdf.aspx?inline=true
7. Australian Institute of Health and Welfare (2010) *National best practice guidelines for collecting Indigenous status in health data sets* https://www.aihw.gov.au/getmedia/ad54c4a7-4e03-4604-a0f3-ccb13c6d4260/11052.pdf.aspx?inline=true
8. Australian Institute of Health and Welfare (2012). *National best practice guidelines for data linkage activities relating to Aboriginal and Torres Strait Islander people*. https://www.aihw.gov.au/getmedia/6d6b9365-9cc7-41ee-873f-13e69e038337/13627.pdf.aspx?inline=true
9. Australian Institute of Health and Welfare (2013). *Taking the next steps: Identification of Aboriginal and Torres Strait Islander status in general practice*. https://www.aihw.gov.au/getmedia/6c8e19b9-2f9d-4ede-8e1f-bd63409ab1b7/15599.pdf.aspx?inline=true
10. Australian Institute of Health and Welfare (2012) *An enhanced mortality database for estimating Indigenous life expectancy: A feasibility study*. https://www.aihw.gov.au/getmedia/3ae4bae6-0777-4faf-acd2-7f3c6e25fe8c/12664.pdf.aspx?inline=true
11. Australian Institute of Health and Welfare (2013). *The inclusion of Indigenous status on pathology request forms*. https://www.aihw.gov.au/getmedia/33d85a0d-6e57-47c5-b9e7-bba313dcfc5c/15247.pdf.aspx?inline=true
12. Australian Institute of Health and Welfare (2013) *Towards better Indigenous health data* (pp. 1–78). https://www.aihw.gov.au/getmedia/b424d472-2e23-4624-a669-22c7e1180036/15020.pdf.aspx?inline=true
13. Australian Institute of Health and Welfare (2013) *Indigenous identification in hospital separations data: Quality report* https://www.aihw.gov.au/getmedia/adcaf32e-d2d1-4df0-b306-c8db7c63022e/13630.pdf.aspx?inline=true
14. Canadian Institute for Health Information (2021) *A path forward: Toward respectful governance of First Nations, Inuit and Métis data housed at CIHI*. https://www.cihi.ca/sites/default/files/document/path-toward-respectful-governance-fnim-2020-report-en.pdf
15. Canadian Institute for Health Information (2022) *Race-based and Indigenous identity data collection and health reporting in Canada*

*supplementary report.*https://www.cihi.ca/sites/default/files/document/race-based-and-indigenous-identity-data-supplementary-report-en.pdf
16. Canadian Partnership Against Cancer (2012) *Environmental scan and analysis of existing patient identification systems for First Nations, Inuit, and Métis peoples* (pp. 1–122). https://s22457.pcdn.co/wp-content/uploads/2018/12/patient-identification-FNIM-2012-EN.pdf
17. Canadian Partnership Against Cancer (2012) *Inventory of profiles: Existing patient identification systems with ethnocultural identifiers specific to First Nations, Inuit, and Métis peoples in Canada*, https://s22457.pcdn.co/wp-content/uploads/2018/12/Inventory-of-Profiles-Existing-Patient-Identification-Systems-with-Ethnocultural-Identifiers-Specific-to-First-Nations-Inuit-and-Métis-EN.pdf
18. Cormack, D. (2007). *Making ethnicity data count* (pp. 44–49). Best Practice Advocacy Centre New Zealand. https://bpac.org.nz/BPJ/2007/October/docs/bpj9_ethnicity_pages_44-46.pdf
19. Cormack, D., & Harris, R. (2009). *Issues in monitoring Māori health and ethnic disparities: An update* (pp. 1–47). Te Rōpū Rangahau Hauora a Eru Pōmare. https://www.otago.ac.nz/wellington/otago600097.pdf
20. Cormack, D., & McLeod, M. (2010). *Improving and maintaining quality in ethnicity data collections in the health and disability sector* (pp. 1–76). Te Rōpū Rangahau Hauora a Eru Pōmare. https://www.otago.ac.nz/wellington/otago600098.pdf
21. Cormack, D., & Robson, C. (2010). *Classification and output of multiple ethnicities: Considerations for monitoring Māori health* (pp. 1–58). Te Rōpū Rangahau Hauora a Eru Pōmare, https://www.fmhs.auckland.ac.nz/assets/fmhs/Te%20Kupenga%20Hauora%20Māori/docs/classification.pdf
22. Government of Canada (2021). *What we heard: Indigenous peoples and COVID-19* https://www.canada.ca/content/dam/phac-aspc/documents/corporate/publications/chief-public-health-officer-reports-state-public-health-canada/from-risk-resilience-equity-approach-covid-19/indigenous-peoples-covid-19-report/cpho-wwh-report-en.pdf
23. International Work Group for Indigenous Affairs (2015). *Indigenous peoples major group position paper on proposed SDG indicators* https://www.iwgia.org/images/publications/0724_SDG_Indicators_Final_eb.pdf
24. First Nations Information Governance Centre. (2020) *A First Nations data governance strategy: A response to direction received from First Nations leadership.* https://fnigc.ca/wp-content/uploads/2020/09/FNIGC_FNDGS_report_EN_FINAL.pdf
25. First Nations Health Authority (2019). *Data and information governance: Case study report* (pp. 1–35). https://www.fnha.ca/Documents/FNHA-BC-Tripartite-Agreement-Case-Study-Data-and-Information-Governance.pdf
26. First Nations Health Authority (n.d.). *First Nations data governance discussion sheet* (pp. 1–2). (n.d.). https://www.fnha.ca/Documents/FNHA-First-Nations-Data-Governance-Discussion-Sheet.pdf
27. Health Canada (2019). *Health Canada data strategy* (pp. 1–47). (2019). https://publications.gc.ca/collections/collection_2021/sc-hc/H14-336-2019-eng.pdf
28. International Group for Indigenous Health Measurement (2008). *Terms of Reference.* https://www.cdc.gov/nchs/data/isp/IGIHM_Terms_of_Reference.pdf
29. Fox, P. (2018). *Indigenous health indicators: A participatory approach to co-designing indicators to monitor and measure First Nations*

health. The Alberta First Nations Information Governance Centre. https://www.afnigc.ca/main/includes/media/pdf/digital%20reports/Indigenous%20Health%20Indicators.pdf
30. Health Statistics Branch: Queensland Health (2015) *Collection of Indigenous status in Queensland health data sets.* https://www.health.qld.gov.au/__data/assets/pdf_file/0029/147629/factsheet1.pdf
31. McBride, K. (n.d.). *Data resources and challenges for First Nations communities: Document review and position paper*. The Alberta First Nations Governance Centre. https://www.afnigc.ca/main/includes/media/pdf/digital%20reports/Data_Resources_Report.pdf
32. Ministry of Health, New Zealand (2004) *Ethnicity data protocols for the health and disability sector* (pp. 1–32). (2004). https://www.fmhs.auckland.ac.nz/assets/fmhs/faculty/tkhm/tumuaki/docs/ethnicity-data-protocols.pdf
33. Ministry of Health, New Zealand (2017) *Ethnicity data protocols* (pp. 1-37). https://www.health.govt.nz/system/files/documents/publications/hiso_10001-2017_ethnicity_data_protocols_21_apr.pdf
34. MMIWGS2S. (2021) *Creating new pathways for data: The 2021 national action plan data strategy* (pp. 1–80). https://mmiwg2splus-nationalactionplan.ca/wp-content/uploads/2021/06/The-2021-National-Action-Plan-Data-Strategy_EN.pdf
35. National Aboriginal Community Controlled Health Organisations (2019). *Submission in response to the office of the national data commissioner data sharing and release discussion paper* (pp. 1–10). https://www.datacommissioner.gov.au/sites/default/files/2019-11/58_0.pdf
36. National Collaborating Centre for Aboriginal Health (2009). *The importance of disaggregated data*. https://www.nccih.ca/docs/context/FS-ImportanceDisaggregatedData-EN.pdf
37. Newfoundland and Labrador (2017). *Newfoundland and Labrador Indigenous administrative data identifier standard.* https://www.mmiwg-ffada.ca/wp-content/uploads/2019/05/40-NL_Indigenous_Administrative_Data_Identifier_Standard_FINAL__2017-12-12.pdf
38. Niagara Region Public Health (2021). *Niagara priority profiles—Indigenous.* https://www.niagararegion.ca/health/equity/pdf/priority-profile-indigenous.pdf
39. NSW Ministry of Health (2012) *Improved reporting of aboriginal and Torres Strait Islander peoples on population datasets in New South Wales using record linkage—a feasibility study*. https://www.health.nsw.gov.au/hsnsw/Publications/atsi-data-linkage-report.pdf
40. Our Health Counts (2018). *Our Health Counts Toronto: An inclusive community-driven health survey for Indigenous peoples in Toronto.* http://www.welllivinghouse.com/wp-content/uploads/2018/02/Mental-Health-OHC-Toronto.pdf
41. Our Health Counts (2017). *Our Health Counts: Urban Indigenous health database project*. Tungasuvvingat Inuit. http://tungasuvvingatinuit.ca/wp-content/uploads/2017/12/Our-Health-Counts-Urban-Indigenous-Health-Database-Project-Inuit-Adults-July-2017.pdf
42. Our Health Counts. *Urban Aboriginal health database research project* (2014). Our Health Counts. http://www.welllivinghouse.com/wp-content/uploads/2014/04/our-health-counts-report-Hamilton2.pdf

43. Province of Manitoba (2021) *COVID-19 infections in Manitoba: Race, ethnicity, and Indigeneity* https://www.gov.mb.ca/health/publichealth/surveillance/docs/rei_external.pdf
44. RACGP National Faculty of Aboriginal and Torres Strait Islander Health. (n.d.) *Identification of Aboriginal and Torres Strait Islander people in Australian general practice* (pp. 1–6). https://www.racgp.org.au/FSDEDEV/media/documents/Faculties/ATSI/Identification-of-Aboriginal-and-Torres-Strait-Islander-people-in-Australian-general-practice.pdf
45. Treventhan, S. (2019). Strengthening the availability of First Nations Data. *Indigenous Services Canada & The Assembly of First Nations.* https://www.afn.ca/wp-content/uploads/2019/05/NCR-11176060-v1-STRENGTHENING_THE_AVAILABILITY_OF_FIRST_NATIONS_DATA-MAR_25_2019-FINAL_E.pdf
46. Tui'kn Partnership (2020) *Overview of the Nova Scotia Mi'kmaw Client Linkage Registry.* *http://www.tuikn.ca/wp-content/uploads/2021/02/Overview-of-the-NSMCLR-Jan-2021.pdf*
47. Queensland Health (2010). *Making tracks toward closing the gap in health outcomes for Indigenous Queenslanders by 2023: Policy and accountability framework.* https://www.health.qld.gov.au/__data/assets/pdf_file/0030/159852/making_tracks_pol.pdf
48. Urban Indian Health Institute (2020) *Best practices for American Indian and Alaska Native data collection*. https://aipi.asu.edu/sites/default/files/best-practices-for-american-indian-and-alaska-native-data-collection.pdf
49. Wilson, G., Willis, J., Totterdell, J., Gupta, A., Chong, A., Clarke, A., Winters, M., Donohue, K., & Posenelli, S. (2017). *Aboriginal identification in hospitals quality improvement program evaluation findings* (pp. 1–16). NSW Ministry of Health. https://www.health.nsw.gov.au/research/Publications/aihqip-evaluation-report.pdf
